# LepRb^+^ cell–specific deletion of *Slug* mitigates obesity and nonalcoholic fatty liver disease in mice

**DOI:** 10.1172/JCI156722

**Published:** 2023-02-15

**Authors:** Min-Hyun Kim, Yuan Li, Qiantao Zheng, Lin Jiang, Martin G. Myers, Wen-Shu Wu, Liangyou Rui

**Affiliations:** 1 Department of Molecular & Integrative Physiology,; 2 Division of Metabolism and Endocrinology, Department of Internal Medicine, University of Michigan Medical School, Ann Arbor, Michigan, USA.; 3 Division of Hematology/Oncology, Department of Medicine, University of Illinois Cancer Center, University of Illinois at Chicago, Chicago, Illinois, USA.; 4 Division of Gastroenterology and Hepatology, Department of Internal Medicine, University of Michigan Medical School, Ann Arbor, Michigan, USA.

**Keywords:** Cell Biology, Metabolism, Diabetes, Leptin, Obesity

## Abstract

Leptin exerts its biological actions by activating the long-form leptin receptor (LepRb). LepRb signaling impairment and leptin resistance are believed to cause obesity. The transcription factor Slug — also known as Snai2 — recruits epigenetic modifiers and regulates gene expression by an epigenetic mechanism; however, its epigenetic action has not been explored in leptin resistance. Here, we uncover a proobesity function of neuronal Slug. Hypothalamic Slug was upregulated in obese mice. LepRb^+^ cell–specific *Slug*-knockout (*Slug*^ΔLepRb^) mice were resistant to diet-induced obesity, type 2 diabetes, and liver steatosis and experienced decreased food intake and increased fat thermogenesis. Leptin stimulated hypothalamic Stat3 phosphorylation and weight loss to a markedly higher level in *Slug*^ΔLepRb^ than in *Slug^fl/fl^* mice, even before their body weight divergence. Conversely, hypothalamic LepRb^+^ neuron–specific overexpression of Slug, mediated by AAV-hSyn-DIO-Slug transduction, induced leptin resistance, obesity, and metabolic disorders in mice on a chow diet. At the genomic level, Slug bound to and repressed the *LepRb* promoter, thereby inhibiting *LepRb* transcription. Consistently, Slug deficiency decreased methylation of *LepRb* promoter H3K27, a repressive epigenetic mark, and increased *LepRb* mRNA levels in the hypothalamus. Collectively, these results unravel what we believe to be a previously unrecognized hypothalamic neuronal Slug/epigenetic reprogramming/leptin resistance axis that promotes energy imbalance, obesity, and metabolic disease.

## Graphical abstract

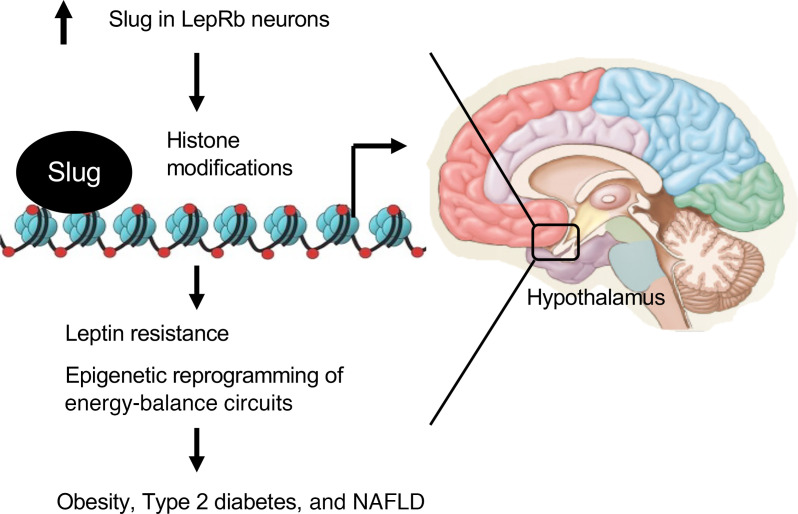

## Introduction

Hypothalamic neural circuits play an essential role in the control of energy balance, body weight, and metabolic homeostasis. GWAS reveal that approximately 95% of human obesity-associated genes and pathways are related to the CNS (1), indicating that hypothalamus and brain dysfunctions are a primary risk factor for obesity and metabolic disease. Leptin is secreted from adipose tissues to relay information about peripheral energy storage and availability to the hypothalamus, and it promotes weight loss by decreasing food intake (2). It also stimulates a sympathetic nerve and fat thermogenesis axis to increase energy expenditure (3, 4). Leptin is believed to exert its metabolic action by activating the long-form leptin receptor LepRb in the hypothalamus (5). In addition to suppressing feeding behavior, hypothalamic LepRb signaling, enhanced by Sh2b1, increases sympathetic nerve outflows to brown adipose tissue (BAT) to promote fat thermogenesis and energy expenditure (3). Impaired leptin action, referred to as leptin resistance, is an important risk for obesity, and leptin resistance impedes leptin therapy to combat obesity and related metabolic disease (2, 4). Hence, molecular mechanisms underlying leptin resistance have gained increased attention. Both negative and positive regulators of LepRb signaling have been identified, and negative and positive regulator imbalances have been proposed to drive leptin resistance (4, 6). Hypothalamic LepRb is downregulated in rodents with high fat diet–induced (HFD-induced) obesity, exacerbating leptin resistance (7–9). Of note, leptin resistance is persistent in diet-induced obesity, raising the possibility that epigenetic reprogramming may be a causal factor for leptin resistance. However, epigenetic modifications are poorly understood in LepRb neural circuits.

Transcription factor Slug, also called Snail2 or Snai2, is a Snail family member — along with Snail1 and Snail3 — that controls gene expression by an epigenetic mechanism (10). Slug binds to target enhancers and promoters — E2 boxes: CACCTG or CAGGTG — via its C-terminal zinc finger domains, while its N-terminal SNAG domain recruits histone deacetylase 1 (HDAC1), HDAC2, lysine-specific demethylase 1 (LSD1), G9a, and/or enhancer of zeste homologue 2 (EZH2) to catalyze histone modifications on target chromatins (10–12). Slug and Snail1 have been well known to promote epithelial-mesenchymal transition (EMT) by epigenetically suppressing E-cadherin expression (10–12). Hepatic Slug promotes liver steatosis and nonalcoholic fatty liver disease (NAFLD) by epigenetically activating lipogenic genes (13). In this work, we report that Slug is expressed in a subset of hypothalamic neurons and is upregulated in obesity. LepRb cell-specific deletion of *Slug* protects against diet-induced leptin resistance, obesity, type 2 diabetes, and NAFLD. Conversely, mediobasal hypothalamus (MBH) LepRb neuron-specific overexpression of Slug has the opposite effects. At the molecular level, Slug binds to the *LepRb* promoter and induces repressive histone methylations, thereby suppressing LepRb expression. These observations suggest hypothalamic LepRb-neuron Slug as a previously unrecognized epigenetic inducer of leptin resistance and obesity.

## Results

### Slug is expressed in a subset of hypothalamic neurons and upregulated in obesity.

**Figure 1:**
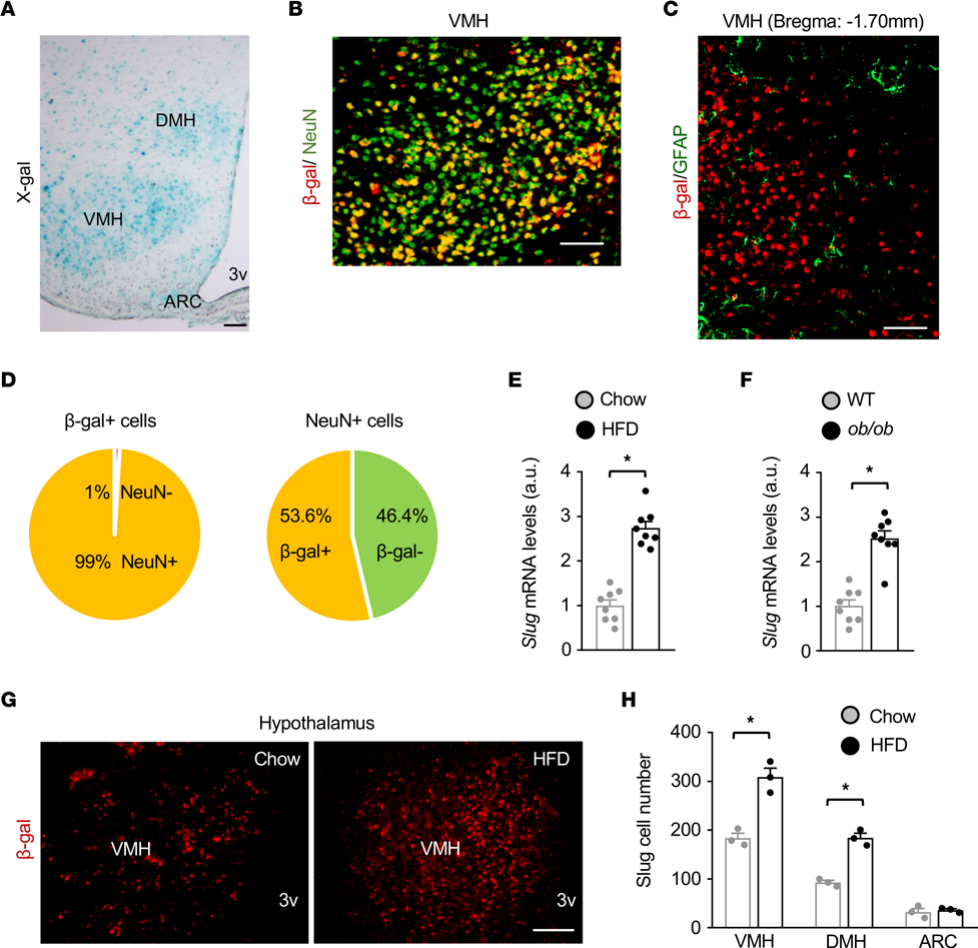
Hypothalamic Slug is upregulated in obesity. (**A**) Representative X-gal staining of *Slug^LacZ/+^* mouse hypothalamic sections (n = 7 mice). (**B** and **C**) Representative hypothalamic images (n = 3 mice per group). Hypothalamic sections were prepared from *Slug^LacZ/+^* mice and coimmunostained with antibodies to β-gal, NeuN, and GFAP as indicated. (**D**) VMH cell subpopulations were counted (n = 3 mice). (**E**) Hypothalamic *Slug* mRNA levels (normalized to 36B4 expression) in C57BL/6J male mice on a HFD for 15 weeks (n = 8 mice per group). a.u., arbitrary units. (**F**) Hypothalamic *Slug* mRNA levels in WT and *ob/ob* male mice at 14 weeks of age (n = 8 mice per group). (**G** and **H**) Hypothalamic sections were prepared from *Slug^LacZ/+^* male mice (HFD for 3 weeks) and stained with anti–β-gal antibody. β-gal neurons in the VMH, DMH, and ARC were counted (n = 3 mice per group). Data are presented as mean ± SEM. *P < 0.05, 2-tailed unpaired Student’s *t* test.

To explore Slug in the brain, we mapped Slug neuron distributions in the hypothalamus. Since anti-Slug antibodies were unable to detect endogenous Slug by immunostaining, we exploited Slug-LacZ reporter mice (*Slug^LacZ^*) in which a β*-galactosidase* (β-gal) transgene was inserted into the *Slug* locus under the control of the *Slug* promoter (14). Slug-expressing cells (β-gal^+^) can be readily detected in heterozygous *Slug^LacZ/+^* mice by X-gal or anti–β-gal–antibody staining (14, 15). We detected abundant β-gal^+^ Slug-expressing cells in the dorsomedial hypothalamus (DMH), ventromedial hypothalamus (VMH), and arcuate nucleus (ARC) (Figure 1A). By contrast, Slug-expressing cells were barely detectable in the cerebral cortex, hippocampus, and cerebellum (Supplemental Figure 1A; supplemental material available online with this article; https://doi.org/10.1172/JCI156722DS1). Hypothalamic Slug-expressing cells coexpressed the neuronal marker NeuN but not the astrocyte marker glial fibrillary acidic protein (GFAP) (Figure 1, B and C). In the VMH, approximately 99% of Slug-expressing cells were neurons, and Slug^+^ neurons accounted for approximately 50% of the total neurons (Figure 1D).

To test if Slug expression was influenced by nutritional states and body weight, we placed C57BL/6J male mice on a HFD for 15 weeks. Hypothalamic *Slug* mRNA levels were significantly higher in mice fed a HFD than in chow-fed mice (Figure 1E), and were also significantly higher in *ob/ob* mice compared with age-matched WT mice (Figure 1F). In contrast, HFD feeding did not increase *Slug* mRNA abundance in the cerebral cortex and cerebellum (Supplemental Figure 1B). To extend these findings, we counted Slug^+^ neurons in the hypothalamus. We placed *Slug^LacZ/+^* mice on a HFD for 3 weeks and immunostained hypothalamic sections with anti-β-gal antibody. Slug^+^ neuron number was significantly higher in the VMH and DMH of HFD-fed mice compared with chow-fed mice (Figure 1, G and H). Given that de novo neurogenesis is rare, the newly generated Slug^+^ neurons in HFD-fed mice may have arisen from neurons that were originally Slug^–^ — a Slug^–^ to Slug^+^ phenotype switch or transdifferentiation.

### LepRb^+^ cell-specific ablation of Slug protects against diet-induced obesity.

**Figure 2:**
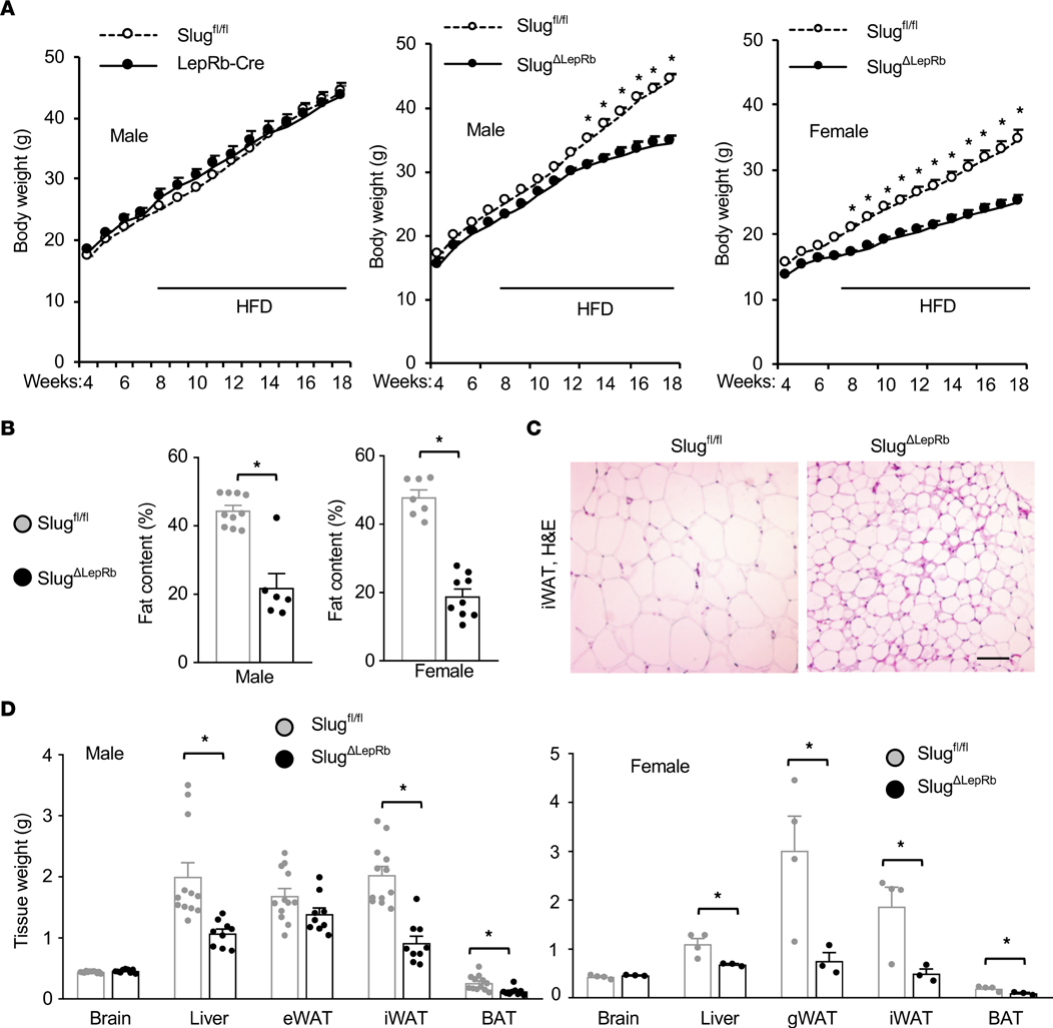
LepRb cell-specific ablation of Slug protects against HFD-induced obesity. (**A**) Growth curves. Left: *Slug^fl/fl^*, n = 24; *LepRb-Cre*, n = 8. Middle: *Slug^fl/fl^*, n = 24; *Slug^ΔLepRb^*, n = 15. Right: *Slug^fl/fl^*, n = 15; *Slug^ΔLepRb^*, n = 13. (**B**) Fat content (% body weight, HFD for 12 weeks). Male *Slug^fl/fl^*, n = 10; male *Slug^ΔLepRb^*, n = 6; female *Slug^fl/fl^*, n = 7; female *Slug^ΔLepRb^*, n = 9. (**C**) Representative H&E staining of male iWAT sections (HFD for 13 weeks, n = 3 mice per genotype). Scale bar: 200 μm. (**D**) Tissue weight (HFD for 13 weeks). Male *Slug^fl/fl^*, n = 12; male *Slug^ΔLepRb^*, n = 9; female *Slug^fl/fl^*, n = 4; female *Slug^ΔLepRb^*, n = 3. Data are presented as mean ± SEM. *P < 0.05, 2-way ANOVA (**A**) and 2-tailed unpaired Student’s *t* test (**B** and **D**).

We set out to investigate hypothalamic Slug functions by generating and characterizing conditional *Slug* knockout mice. Considering the essential role of LepRb^+^ neurons in the control of body weight, we generated LepRb^+^ cell-specific *Slug* knockout (*Slug^ΔLepRb^*) mice by crossing *Slug^fl/fl^* mice with *LepRb-Cre* mice. *Slug^fl/fl^* mice and *LepRb-Cre* mice — with *Cre* knockin at the *LepRb* locus — were described previously (13, 16). In *LepRb-Cre* mice, Cre is expressed in cells expressing LepRb but not in cells expressing short forms of leptin receptors (16). We validated *Slug^ΔLepRb^* mice using RNAscope assays. *Slug* probes were designed to hybridize mRNA fragments encoded by the deleted *Slug* sequences in *Slug^ΔLepRb^* mice. We detected hypothalamic Slug^+^LepRb^+^ double-positive neurons in *Slug^fl/fl^* but not *Slug^ΔLepRb^* mice at 8 weeks of age on chow diet (Supplemental Figure 2A). Slug^+^LepRb^+^ neuron number was underestimated because the *Slug* probes were unable to detect Slug^+^ neurons expressing Slug at low levels. Additionally, Slug^+^LepRb^+^ neurons were expected to be increased in obesity, given that HFD feeding increases hypothalamic Slug^+^ neuron number. We placed *Slug^ΔLepRb^*, *Slug^fl/fl^*, and *LepRb-Cre* mice on a HFD. Body weight was indistinguishable between *Slug^fl/fl^* and *LepRb-Cre* mice (Figure 2A), so *Slug^fl/fl^* mice were used as a control in the following experiments. Both male and female *Slug^ΔLepRb^* mice were markedly resistant to HFD-induced obesity, and their body weights were significantly lower compared with sex-and age-matched *Slug^fl/fl^* mice (Figure 2A). Whole-body fat content was substantially lower in *Slug^ΔLepRb^* than in *Slug^fl/fl^* mice on a HFD for 12 weeks (Figure 2B). Lean mass was comparable in *Slug^ΔLepRb^* and *Slug^fl/fl^* mice (Supplemental Figure 2B). Individual white adipocyte size was smaller in *Slug^ΔLepRb^* than in *Slug^fl/fl^* mice (Figure 2C). Inguinal white adipose tissue (iWAT), BAT, and liver weights were significantly lower in *Slug^ΔLepRb^* than in *Slug^fl/fl^* males on a HFD for 13 weeks (Figure 2D). Likewise, iWAT, gonadal WAT (gWAT), BAT, and liver weights were also markedly lower in *Slug^ΔLepRb^* than in *Slug^fl/fl^* females (Figure 2D). We also examined *Slug^ΔLepRb^* mice on a normal chow diet. Body weight was comparable between *Slug^ΔLepRb^* and *Slug^fl/fl^* mice at 8 weeks of age, but thereafter, *Slug^ΔLepRb^* mice progressively gained less body weight and, after 12 weeks of age, became significantly lighter than *Slug^ΔLepRb^* mice (Supplemental Figure 2C). Epididymal WAT (eWAT) weight was significantly lower in *Slug^ΔLepRb^* than in *Slug^fl/fl^* mice at 13 weeks of age (Supplemental Figure 2D). These results demonstrate, for the first time to our knowledge, that LepRb^+^ cell-specific Slug deficiency protects against both diet-induced and age-associated obesity.

### LepRb^+^ cell-specific ablation of Slug protects against HFD-induced type 2 diabetes and NAFLD.

**Figure 3:**
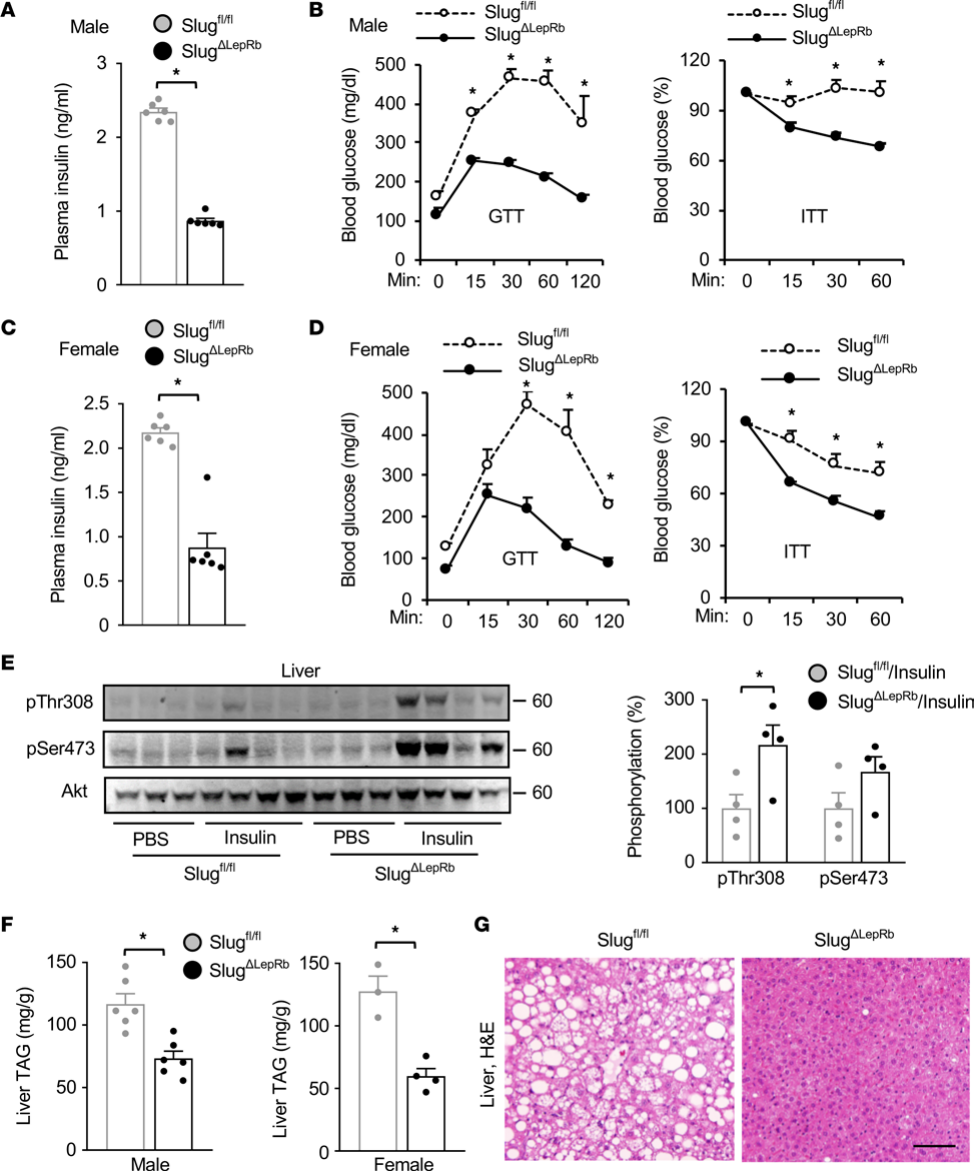
LepRb cell–specific ablation of Slug protects against HFD-induced type 2 diabetes and NAFLD. (**A**) Male overnight-fasted plasma insulin levels (HFD for 10 weeks). *Slug^fl/fl^*, n = 6; *Slug^ΔLepRb^*, n = 6. (**B**) Male GTT and ITT (HFD for 13 weeks). *Slug^fl/fl^*, n = 4; *Slug^ΔLepRb^*, n=5. (**C**) Female overnight-fasted plasma insulin levels (HFD for 10 weeks). *Slug^fl/fl^*, n = 6; *Slug^ΔLepRb^*, n = 6. (**D**) Female GTT and ITT (HFD for 12 weeks). *Slug^fl/fl^*, n = 3; *Slug^ΔLepRb^*, n = 4. (**E**) *Slug^fl/fl^* and *Slug^ΔLepRb^* males (HFD for 13 weeks) were fasted overnight and treated with PBS (n = 3) or insulin (n = 4). Liver extracts were immunoblotted with antibodies to phospho-Akt and Akt. Phospho-Akt was normalized to Akt. (**F**) Liver TAG levels (normalized to liver weight; HFD for 13 weeks). Male *Slug^fl/fl^*, n = 6; male *Slug^ΔLepRb^*, n = 6; female *Slug^fl/fl^*, n = 3; female *Slug^ΔLepRb^*, n = 4. (**G**) Representative H&E staining of male liver sections (HFD for 13 weeks, n = 4 mice per group). Scale bar: 200 μm. Data are presented as mean ± SEM. *P < 0.05, 2-tailed unpaired Student’s *t* test (**A**, **C**, **E**, and **F**) and 2-way ANOVA (**B** and **D**).

We placed *Slug^ΔLepRb^* and *Slug^fl/fl^* mice on a HFD and assessed their glucose metabolism. Overnight-fasted plasma insulin levels were significantly lower in *Slug^ΔLepRb^* than in *Slug^fl/fl^* male mice on a HFD for 10 weeks (Figure 3A). Glucose tolerance tests (GTT) and insulin tolerance tests (ITT) were performed in male mice on a HFD for 13 weeks. Blood glucose levels were significantly lower in *Slug^ΔLepRb^* mice relative to *Slug^fl/fl^* mice after glucose and insulin injections (Figure 3B). Similarly, *Slug^ΔLepRb^* female mice also displayed reduced plasma insulin levels after being fed a HFD for 10 weeks and improved glucose and insulin tolerances after being fed a HFD for 12 weeks, compared with *Slug^fl/fl^* females (Figure 3, C and D). In line with these results, insulin stimulation increased liver Akt phosphorylation to a significantly higher level in *Slug^ΔLepRb^* mice relative to *Slug^fl/fl^* mice on a HFD for 13 weeks (Figure 3E). Akt phosphorylation was low in *Slug^fl/fl^* mice due to HFD-induced insulin resistance. Liver weight (Figure 2D) and liver triacylglycerol (TAG) levels (Figure 3F) were significantly lower in *Slug^ΔLepRb^* than in *Slug^fl/fl^* mice on a HFD for 13 weeks, for both males and females. Hepatocyte lipid droplets were smaller and less abundant in *Slug^ΔLepRb^* mice relative to *Slug^fl/fl^* mice, as revealed by H&E and Nile red staining of liver sections (Figure 3G and Supplemental Figure 2E). We also performed GTT and ITT on chow-fed *Slug^ΔLepRb^* and *Slug^fl/fl^* mice at 8 weeks of age, when their body weights were comparable. GTT and ITT were indistinguishable between *Slug^ΔLepRb^* and *Slug^fl/fl^* mice (Supplemental Figure 2F). Thus, LepRb^+^ cell–specific ablation of Slug protected against diet-induced obesity, type 2 diabetes, and NAFLD.

### LepRb^+^ cell-specific ablation of Slug decreases food intake and increases adipose thermogenesis.

**Figure 4:**
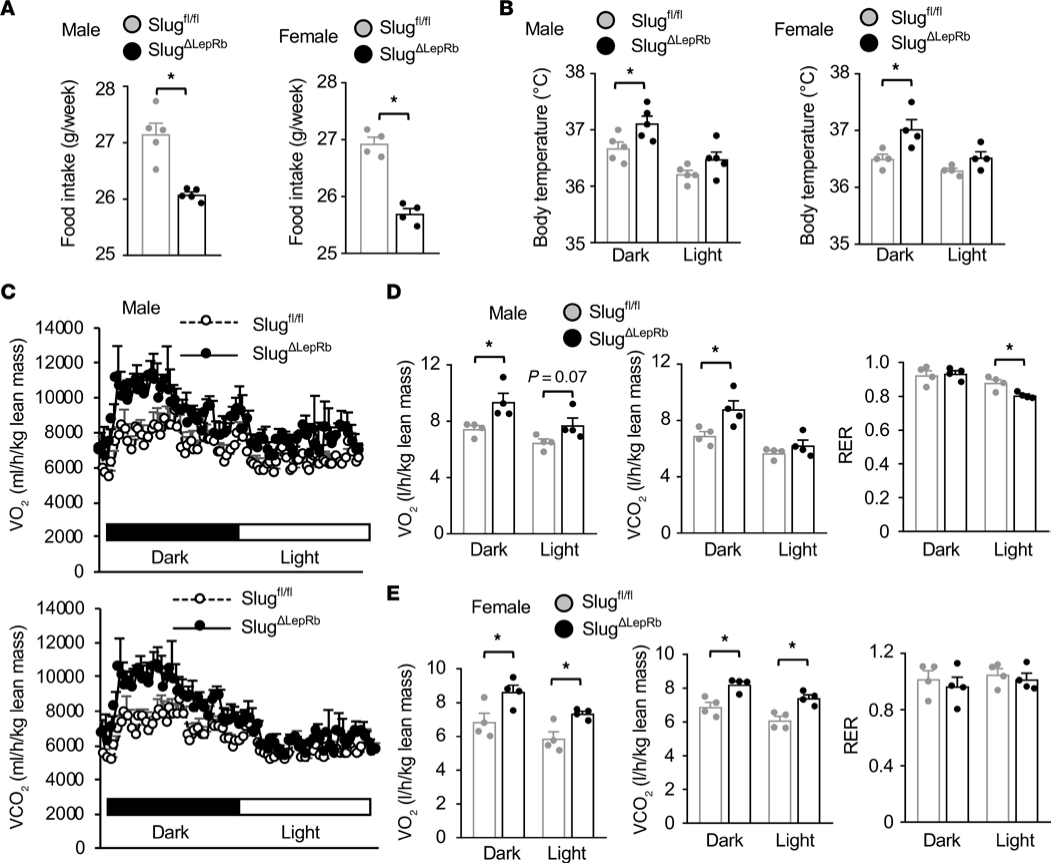
Slug deficiency in LepRb cells induces energy imbalance. Mice were fed a chow diet. (**A**) Food intake at 9 weeks of age. Male *Slug^fl/fl^*, n = 5; male *Slug^ΔLepRb^*, n = 5; female *Slug^fl/fl^*, n = 4; female *Slug^ΔLepRb^*, n = 4. (**B**) Body core temperatures at 9–10 weeks of age. Male *Slug^fl/fl^*, n = 5; male *Slug^ΔLepRb^*, n = 5; female *Slug^fl/fl^*, n = 4; female *Slug^ΔLepRb^*, n = 4. (**C–E**) O_2_ consumption, CO_2_ production (normalized to lean mass), and respiratory exchange ratio (RER) at 9 weeks of age (n = 4 mice per group). Data are presented as mean ± SEM. *P < 0.05, 2-tailed unpaired Student’s *t* test.

We postulated that *Slug^ΔLepRb^* mice might resist obesity through decreasing their food intake, increasing their energy expenditure, or both. Supporting this notion, we found that food intake was substantially lower in *Slug^ΔLepRb^* males and females relative to sex- and age-matched *Slug^fl/fl^* mice (Figure 4A). Body temperature, an energy expenditure index, was significantly higher in *Slug^ΔLepRb^* than in *Slug^fl/fl^* mice in the dark phase (Figure 4B). We assessed energy expenditure using metabolic cages. O_2_ consumption and CO_2_ production, normalized to lean mass, were significantly higher in *Slug^ΔLepRb^* mice relative to *Slug^fl/fl^* mice in both males and females, particularly in the dark phase (Figure 4, C–E). However, ANCOVA calculations did not reveal a significant difference between the 2 groups, possibly due to a low animal number limiting ANCOVA statistical power under this condition.

**Figure 5:**
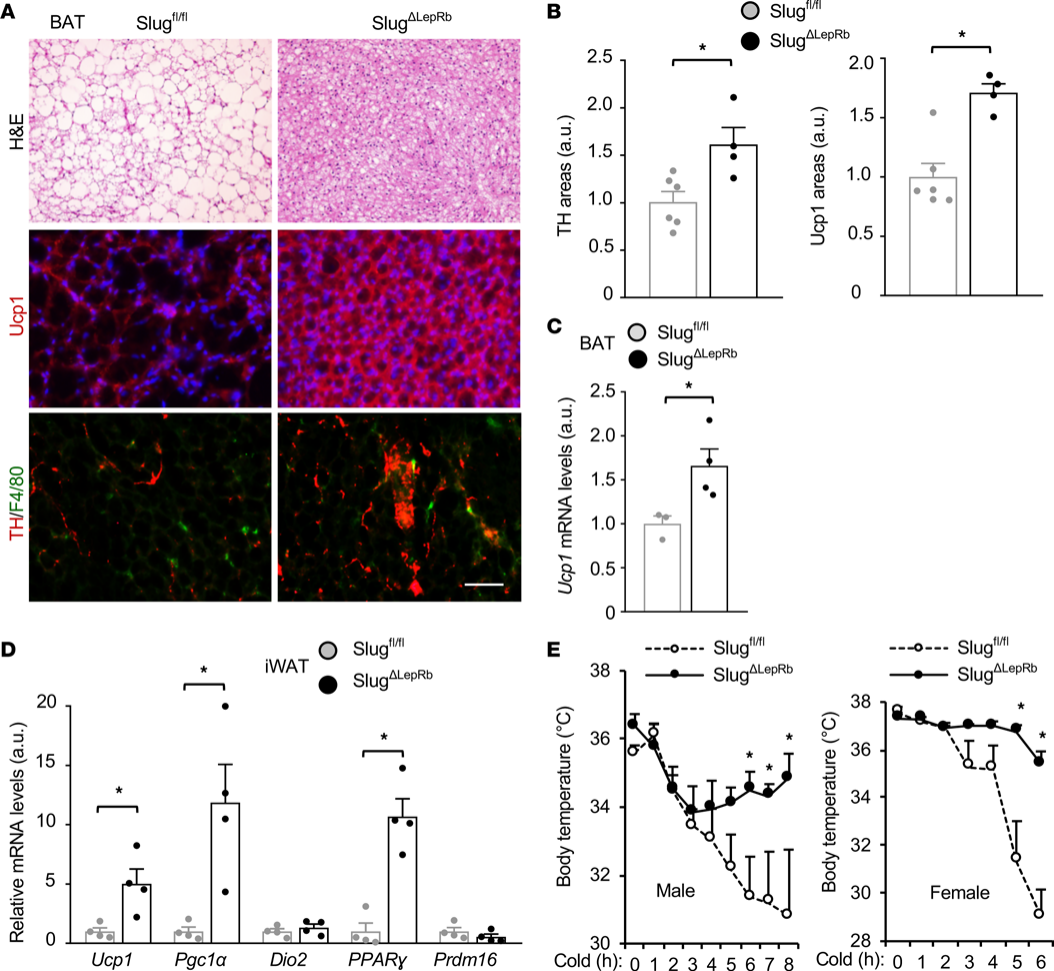
Slug deficiency in LepRb cells enhances adipose thermogenesis. (**A**) Representative BAT images (n = 4 mice per group). BAT sections were stained with H&E (HFD for 15 weeks) or antibodies to Ucp1 and TH (HFD for 12 weeks). Scale bar: 200 μm. (**B**) Ucp1 and TH areas were quantified and normalized to total areas. *Slug^fl/fl^*, n = 6; *Slug^ΔLepRb^*, n = 4. a.u., arbitrary units. (**C**) Male BAT *Ucp1* mRNA levels (normalized to 36B4 expression, HFD for 12 weeks). *Slug^fl/fl^*, n = 3; *Slug^ΔLepRb^*, n = 4. (**D**) Gene expression in iWAT (normalized to 36B4 levels). Male mice (10 weeks) were exposed to cold (8°C for 2 hours) daily for 5 days. *Slug^fl/fl^*, n = 4; *Slug^ΔLepRb^*, n = 4. (**E**) Cold tolerance test at 19 weeks of age on chow diet. Male *Slug^fl/fl^*, n = 4; male *Slug^ΔLepRb^*, n = 4; female *Slug^fl/fl^*, n = 4; female *Slug^ΔLepRb^*, n = 5. Data are presented as mean ± SEM. *P < 0.05, 2-tailed unpaired Student’s *t* test (**B–D**) and 2-way ANOVA (**E**).

BAT and beige fat protect against obesity by increasing energy expenditure (17), prompting us to examine adipose thermogenic programs in *Slug^ΔLepRb^* mice. We placed *Slug^fl/fl^* and *Slug^ΔLepRb^* mice on a HFD for 12–15 weeks. HFD feeding induced whitening of BAT in *Slug^fl/fl^* mice, as illustrated by enlarged lipid droplets (Figure 5A). Remarkably, *Slug^ΔLepRb^* mice were completely resistant to HFD-induced BAT whitening (Figure 5A). BAT Ucp1 protein and mRNA levels were significantly higher in *Slug^ΔLepRb^* than in *Slug^fl/fl^* mice (Figure 5, A–C). Sympathetic nerve inputs are known to increase BAT thermogenesis. Sympathetic nerve innervations, as assessed by immunostaining of BAT sections with antibody against the sympathetic nerve marker tyrosine hydroxylase (TH), were significantly higher in *Slug^ΔLepRb^* than in *Slug^fl/fl^* mice (Figure 5, A and B). *Slug^ΔLepRb^* mice displayed increased recruitment of beige adipocytes; iWAT expression of beige adipocyte markers — Ucp1, Pgc1α, and PPARγ — was significantly higher in *Slug^ΔLepRb^* than in *Slug^fl/fl^* mice after 5 days of cold exposure (Figure 5D). To assess adaptive thermogenesis in vivo, we placed *Slug^ΔLepRb^* mice at ambient cold temperature, 4^0^C, and monitored body core temperature through the rectum. Body core temperature was considerably higher in *Slug^ΔLepRb^* mice relative to *Slug^fl/fl^* mice following cold exposure (Figure 5E). These results unveil what we believe to be a previously unrecognized hypothalamic Slug/sympathetic nerve/thermogenic fat axis.

### MBH LepRb^+^ neuron-specific overexpression of Slug induces obesity and metabolic disorders.

**Figure 6:**
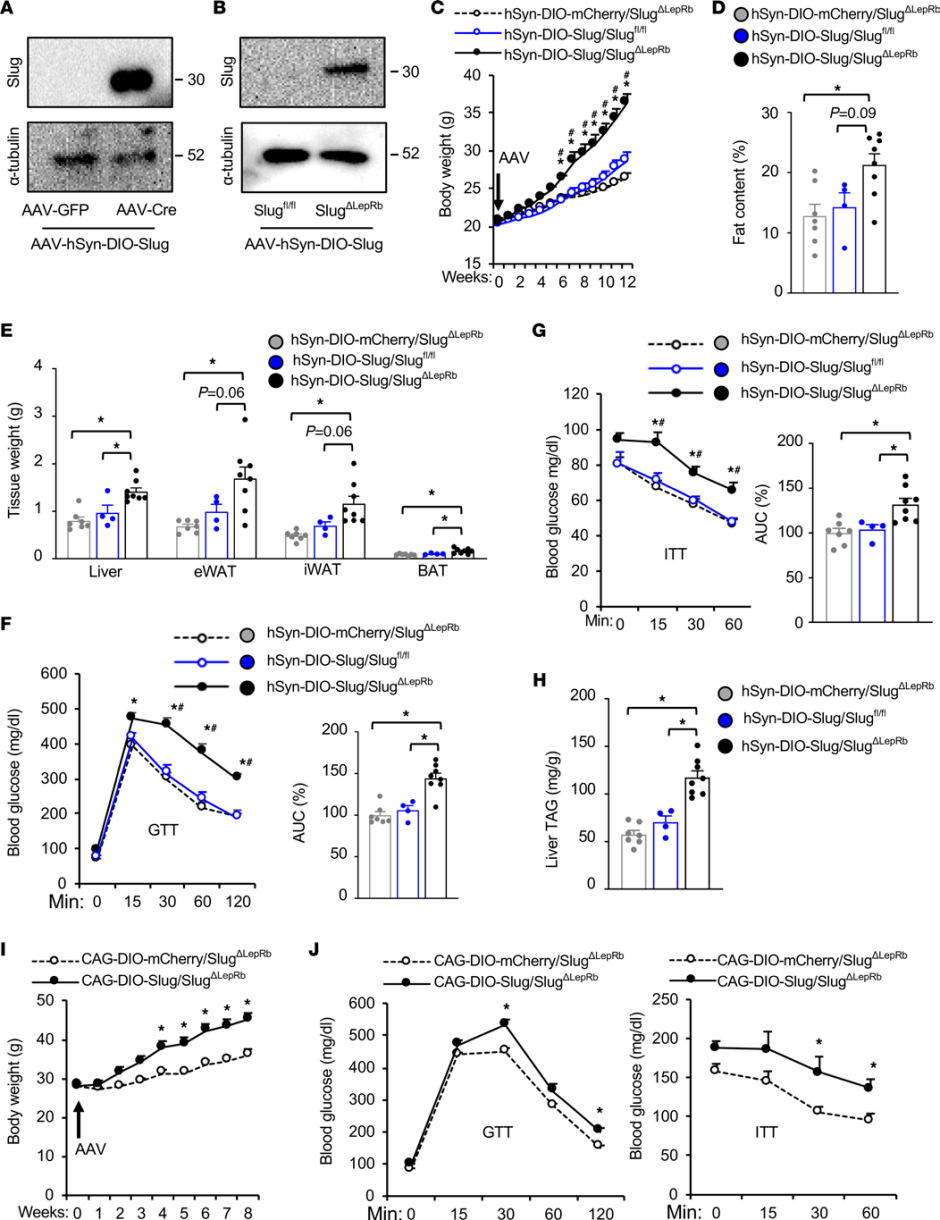
Hypothalamic LepRb neuron–specific overexpression of Slug induces obesity. (**A**) AAV-hSyn-DIO-Slug vectors were coinjected with either AAV-CAG-GFP or AAV-CAG-Cre vectors into the brains of C57BL/6J mice. Brain extracts were prepared 3 weeks later and immunoblotted with antibodies against Slug or α-tubulin. (**B–H**) AAV-hSyn-DIO-Slug or AAV-hSyn-DIO-mCherry vectors were bilaterally microinjected into the MBH of *Slug^ΔLepRb^* (*Slug^fl/fl^;LepRb-Cre^+/+^*) males (8 weeks, on chow diet). *Slug^fl/fl^* males (lacking *Cre*) were injected with AAV-hSyn-DIO-Slug vectors (control). (**B**) Hypothalamic extracts were immunoblotted with antibodies against Slug or α-tubulin (12 weeks after AAV transduction). (**C**) Body weight. (**D**) Fat content at 12 weeks after AAV transduction (% body weight). (**E**) Tissue weight (12 weeks after AAV transduction). (**F** and **G**) GTT and ITT at 11 weeks after AAV transduction. (**H**) Liver TAG levels at 12 weeks after AAV transduction (normalized to liver weight). AAV-hSyn-DIO-Slug/ *Slug^ΔLepRb^*, n = 8; AAV-hSyn-DIO-mCherry/*Slug^ΔLepRb^*, n = 7; AAV-hSyn-DIO-Slug/*Slug^fl/fl^*, n = 4. (**I** and **J**) AAV-CAG-DIO-Slug or AAV-CAG-DIO-mCherry vectors were bilaterally microinjected into the MBH of *Slug^ΔLepRb^* (*Slug^fl/fl^;LepRb-Cre^+/–^*) male mice at 9 weeks of age on chow diet (n = 5 mice per group). (**I**) Body weight. (**J**) GTT (2 g glucose/kg) and ITT (1 unit insulin/kg) at 8 weeks after AAV transduction. Data are presented as mean ± SEM. *P < 0.05, 1-way (**D** and **E**, **F** [right panel], **G** [right panel], and **H**) and 2-way (**C**, **F** [left panel], **G** [left panel], **I**, and **J**) ANOVA.

*Slug* is expected to be deleted in LepRb-expressing cells in both the brain and peripheral tissues of *Slug^ΔLepRb^* mice. We next examined hypothalamic LepRb–neuron Slug. To test whether MBH LepRb neuron–specific restoration of Slug reversed the obesity-resistant phenotype of *Slug^ΔLepRb^* mice, we generated neuron-specific, Cre-dependent AAV-hSyn-DIO-Slug vectors. The *Slug* cDNA sequences were flanked by 2 loxp sites in a head-to-head orientation, and the double-floxed inverse orientation (DIO) *Slug* transgene was placed under the control of the human *synapsin-1* (hSyn) promoter (Supplemental Figure 3A). Cre-mediated *Slug* orientation reversion activated *Slug* transgene expression. To validate the vector, we comicroinjected AAV-hSyn-DIO-Slug vectors with either AAV-CAG-Cre or AAV-CAG-GFP vectors into mouse brains. Recombinant Slug was detected in AAV-CAG-Cre, but not AAV-CAG-GFP, coinjected brains (Figure 6A), demonstrating Cre-dependent Slug expression. AAV-hSyn-DIO-Slug or AAV-hSyn-DIO-mCherry (control) vectors were bilaterally microinjected into the MBH in *Slug^ΔLepRb^* (i.e., *Slug^fl/fl^;LepRb-Cre*^+/+^) — at 8 weeks old and on a chow diet — or *Slug^fl/fl^* mice (i.e., Cre-negative control). We verified MBH-restricted microinjections (Supplemental Figure 3B). As expected, recombinant Slug was detected in hypothalamic extracts from hSyn-DIO-Slug/*Slug^ΔLepRb^* (i.e., *LepRb-Cre^+/+^*) but not hSyn-DIO-Slug/*Slug^fl/fl^* (i.e., *LepRb-Cre*^–^) mice 12 weeks after AAV transduction (Figure 6B). Of note, recombinant Slug levels in hSyn-DIO-Slug/*Slug^ΔLepRb^* mice were higher than endogenous Slug levels in hSyn-DIO-Slug/*Slug^fl/fl^* mice, which were below detection threshold (Figure 6B). Remarkably, hSyn-DIO-Slug*/Slug^ΔLepRb^* mice — with MBH LepRb^+^ neuron-specific Slug overexpression and on a chow diet — gained substantially more body weight compared with hSyn-DIO-Slug/*Slug^fl/fl^* and hSyn-DIO-mCherry/*Slug^ΔLepRb^* mice after 6 weeks of AAV transduction (Figure 6C). hSyn-DIO-Slug/*Slug^fl/fl^* mice, which express endogenous Slug, were slightly heavier than the *Slug* knockout hSyn-DIO-mCherry/*Slug^ΔLepRb^* mice after 12 weeks of AAV transduction (Figure 6C), supporting the observation that *Slug^ΔLepRb^* mice were resistant to age-associated obesity (Supplemental Figure 2C). Fat content and eWAT, iWAT, and BAT weights were markedly higher in hSyn-DIO-Slug/*Slug^ΔLepRb^* mice relative to hSyn-DIO-mCherry/*Slug^ΔLepRb^* mice and hSyn-DIO-Slug/*Slug^fl/fl^* mice at 12 weeks after AAV transduction (Figure 6, D and E). Lean mass was slightly higher, but lean mass–to–body weight ratios were lower due to increased body weight, in hSyn-DIO-Slug/*Slug^ΔLepRb^* mice (Supplemental Figure 3C). In GTT and ITT, blood glucose levels were significantly higher in hSyn-DIO-Slug/*Slug^ΔLepRb^* mice compared with hSyn-DIO-mCherry/*Slug^ΔLepRb^* mice and hSyn-DIO-Slug/*Slug^fl/fl^* mice (Figure 6, F and G). Liver weights (Figure 6E) and liver TAG levels (Figure 6H) were substantially higher in hSyn-DIO-Slug/*Slug^ΔLepRb^* mice compared with hSyn-DIO-mCherry/*Slug^ΔLepRb^* and hSyn-DIO-Slug/*Slug^fl/fl^* mice. To verify these observations, we generated distinct Cre-dependent AAV-CAG-DIO-Slug vectors by replacing the *hSyn* promoter with the CAG promoter (Supplemental Figure 3D). We confirmed that recombinant Slug expression was dependent on Cre (Supplemental Figure 3E). AAV-CAG-DIO-Slug or AAV-CAG-DIO-mCherry vectors were bilaterally microinjected into the MBH of *Slug^ΔLepRb^* (i.e., *Slug^fl/fl^;LepRb-Cre^+/–^*) male mice at 9 weeks of age. As expected, recombinant Slug was detected in hypothalamic extracts from CAG-DIO-Slug/*Slug^ΔLepRb^* but not CAG-DIO-mCherry/*Slug^ΔLepRb^* mice (Supplemental Figure 3F). Importantly, CAG-DIO-Slug/*Slug^ΔLepRb^* mice gained substantially more body weight than CAG-DIO-mCherry/*Slug^ΔLepRb^* mice after 4 weeks of AAV transduction while on a chow diet (Figure 6I). Fat content was significantly higher in CAG-DIO-Slug/*Slug^ΔLepRb^* than in CAG-DIO-mCherry/*Slug^ΔLepRb^* mice at 9 weeks after AAV transduction, whereas lean mass was comparable between the 2 groups (Supplemental Figure 3G). In GTT and ITT, 8 weeks after AAV transduction, CAG-DIO-Slug/*Slug^ΔLepRb^* mice developed glucose intolerance and insulin resistance compared with CAG-DIO-mCherry/*Slug^ΔLepRb^* mice (Figure 6J). Thus, MBH LepRb^+^ neuron–specific overexpression of Slug is sufficient to induce obesity on a chow diet. We believe that these results unveil hypothalamic LepRb neuron Slug as a previously unrecognized molecular promoter of obesity and metabolic disease.

### Hypothalamic LepRb–neuron Slug promotes leptin resistance.

**Figure 7:**
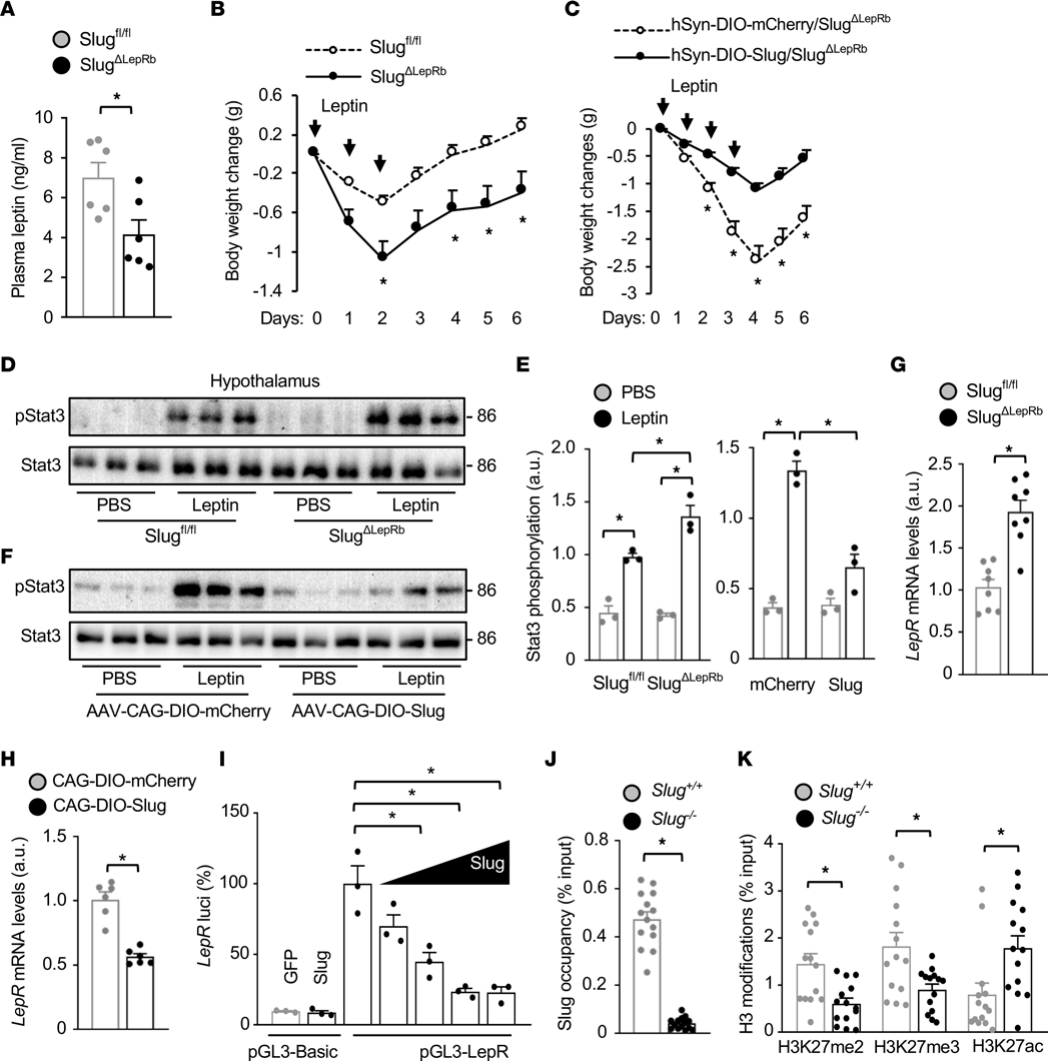
Slug directly suppresses LepRb expression and induces leptin resistance. (**A**) Male overnight-fasted plasma leptin levels (8 weeks old, chow diet, n = 6 mice per group). (**B**) Male mice (7 weeks old, chow diet) were treated with leptin for 3 days. Body weight changes were measured (n = 5 mice per group). (**C**) AAV-hSyn-DIO-Slug (n = 8) or AAV-hSyn-DIO-mCherry (n = 7) vectors were bilaterally microinjected into the MBH of *Slug^ΔLepRb^* male mice (*Slug^fl/fl^; LepRb-Cre^+/+^*) on a chow diet. Twelve weeks later, mice were treated with leptin and body weight changes were measured. (**D** and **E**) *Slug^fl/fl^* and *Slug^ΔLepRb^* males (8 weeks old, chow diet) were fasted overnight and treated with leptin (i.p., 1 mg/kg, 45 minutes). Hypothalamic extracts were immunoblotted with anti–phospho-Stat3 (pTyr705) and anti-Stat3 antibodies. Stat3 phosphorylation was normalized to Stat3 levels (n = 3 mice per group). a.u., arbitrary units. (**F**) AAV-CAG-DIO-Slug or AAV-CAG-DIO-mCherry vectors were bilaterally microinjected into the MBH of *Slug^ΔLepRb^* (*Slug^fl/fl^;LepRb-Cre^+/–^*) males (9 weeks old, on chow diet). Two weeks later, the mice were treated with leptin (i.p., 1.2 mg/kg, 45 minutes). Hypothalamic extracts were immunoblotted with anti–phospho-Stat3 and anti-Stat3 antibodies. Stat3 phosphorylation was normalized to Stat3 levels (**E**, n = 3 mice per group). (**G**) Male hypothalamic *LepR* mRNA levels (normalized to 36B4 levels, 8 weeks old, chow diet, n = 8 mice per group). (**H**) Hypothalamic *LepR* mRNA levels 2 weeks after AAV transduction (normalized to 36B4 levels, n = 6 mice per group). AAV-CAG-DIO-Slug or AAV-CAG-DIO-mCherry vectors were bilaterally microinjected into the MBH of *Slug^ΔLepRb^* (*Slug^fl/fl^;LepRb-Cre^+/–^*) male mice (9 weeks old, chow diet). (**I**) *LepR* promoter luciferase reporter activity (n = 3 per group). (**J** and **K**) Hypothalamic Slug occupancy on the *LepR* promoter (**J**) and hypothalamic *LepR* promoter H3K27me2, H3K3me3, and H3K27ac levels (**K**). *Slug^+/+^* (n = 14) and *Slug^–/–^* (n = 14) males were fed a HFD for 14–16 weeks. Data are presented as mean ± SEM. *P < 0.05, 2-tailed unpaired Student’s *t* test (**A**, **G**, **H**, **J**, and **K**), 1-way (**I**) and 2-way ANOVA (**B**, **C**, and **E**).

We postulated that hypothalamic Slug might promote obesity by inducing leptin resistance. Considering that hyperleptinemia is often associated with leptin resistance, we measured blood leptin levels. Leptin levels were substantially lower in *Slug^ΔLepRb^* males and females relative to sex- and age-matched *Slug^fl/fl^* mice on HFD (Supplemental Figure 4A). To exclude body-weight influence on leptin secretion, we measured leptin levels in *Slug^ΔLepRb^* and *Slug^fl/fl^* mice on a chow diet at 8 weeks of age, when their body weights were comparable. Plasma leptin levels were still significantly lower in *Slug^ΔLepRb^* mice (Figure 7A). To assess leptin sensitivity in vivo, we treated *Slug^ΔLepRb^* and *Slug^fl/fl^* mice on a chow diet at 7 weeks of age — when their body weights were similar — with leptin for 3 days and monitored body weight changes. Leptin treatments decreased body weight to a significantly higher degree in *Slug^ΔLepRb^* mice compared with *Slug^fl/fl^* mice (Figure 7B). To complement these findings, we tested whether MBH LepRb neuron–specific overexpression of Slug inhibits leptin actions. AAV-hSyn-DIO-Slug or AAV-hSyn-DIO-mCherry vectors were bilaterally microinjected into the MBH of *Slug^ΔLepRb^* mice on a chow diet. The AAV-transduced mice were treated with leptin for 4 days, and their body weights were monitored. Leptin decreased body weight to a significantly lesser degree in hSyn-DIO-Slug/*Slug^ΔLepRb^* mice than in hSyn-DIO-mCherry*/Slug^ΔLepRb^* mice (Figure 7C). To corroborate these results, we assessed hypothalamic leptin signaling in these mice. *Slug^ΔLepRb^* and *Slug^fl/fl^* male mice — 8 weeks old and on a chow diet — were fasted overnight and i.p. injected with leptin 45 minutes before hypothalamic extracts were taken and were prepared and immunoblotted with anti–phospho-Stat3 antibody. Body weight (Supplemental Figure 4B) and baseline Stat3 phosphorylation (Figure 7D) were similar between *Slug^ΔLepRb^* and *Slug^fl/fl^* mice, but leptin-stimulated phosphorylation of Stat3 was significantly higher in *Slug^ΔLepRb^* mice (Figure 7, D and E). To extend these findings, we directly injected leptin into the brain of *Slug^ΔLepRb^* and *Slug^fl/fl^* mice at 7 weeks of age and immunostained hypothalamic sections with phospho-Stat3 antibody. The number of phospho-Stat3–positive neurons in the ARC and VMH were significantly higher in *Slug^ΔLepRb^* mice relative to *Slug^fl/fl^* mice (Supplemental Figure 4C). To test if MBH LepRb^+^ neuron-specific overexpression of Slug suppressed leptin signaling, we bilaterally microinjected AAV-CAG-DIO-Slug or AAV-CAG-DIO-mCherry vectors into the MBH of *Slug^ΔLepRb^* (i.e., *Slug^fl/fl^;LepRb-Cre^+/–^*) mice on a chow diet at 9 weeks of age. Two weeks later, the mice were fasted overnight and stimulated with leptin, and hypothalamic extracts were immunoblotted with anti–phospho-Stat3 antibody. Body weight (Supplemental Figure 4D) and baseline Stat3 phosphorylation (Figure 7E) were comparable between CAG-DIO-Slug/*Slug^ΔLepRb^* and CAG-DIO-mCherry/*Slug^ΔLepRb^* mice, but leptin-stimulated phosphorylation of Stat3 was significantly lower in CAG-DIO-Slug/*Slug^ΔLepRb^* mice (Figure 7, E and F). Taken together, these results demonstrate that LepRb^+^ neuron–intrinsic Slug cell-autonomously suppresses leptin signaling to induce leptin resistance, which leads to obesity.

### Slug directly suppresses LepRb expression by an epigenetic mechanism.

We next set out to identify molecular targets of hypothalamic Slug. We isolated the hypothalamus from *Slug^ΔLepRb^* and *Slug^fl/fl^* mice for Affymetrix GeneChip analysis (GSE217748). We found 180 genes upregulated by more than 1.25-fold (*P* < 0.05) and 70 genes downregulated by more than 25% (*P* < 0.05) in *Slug^ΔLepRb^* mice (Supplemental Figure 5, A and B). These putative targets were annotated to multiple pathways, including leptin signaling pathways (Supplemental Figure 5C). Interestingly, LepRb expression was substantially upregulated in *Slug^ΔLepRb^* mice (Supplemental Figure 5, A and B). By quantitative PCR (qPCR), we confirmed that hypothalamic *LepRb* mRNA levels were significantly higher in *Slug^ΔLepRb^* than in *Slug^fl/fl^* mice that had been on a HFD for 15 weeks (Supplemental Figure 6A). To exclude body-weight influence on LepRb expression, we measured LepRb abundance in *Slug^ΔLepRb^* and *Slug^fl/fl^* mice on chow diet at 8 weeks of age, when their body weights were similar (Supplemental Figure 4B). Hypothalamic *LepRb* mRNA levels were still significantly higher in *Slug^ΔLepRb^* than in *Slug^fl/fl^* mice (Figure 7G). To determine whether LepRb^+^ neuron-specific overexpression of Slug inhibits LepRb expression, we bilaterally microinjected AAV-CAG-DIO-Slug or AAV-CAG-DIO-mCherry vectors into the MBH of *Slug^ΔLepRb^* (i.e., *Slug^fl/fl^;LepRb-Cre^+/–^*) mice at 9 weeks of age on a chow diet and isolated the hypothalamus 2 weeks later. Body weight was comparable between CAG-DIO-Slug/*Slug^ΔLepRb^* and CAG-DIO-mCherry/*Slug^ΔLepRb^* mice (Supplemental Figure 4D), but hypothalamic-*LepRb* mRNA levels were significantly lower in CAG-DIO-Slug/*Slug^ΔLepRb^* mice with MBH LepRb^+^ neuron–specific overexpression of Slug (Figure 7H).

We noticed that both mouse and human *LepR* promoters contain putative Slug response elements in the form of E2 boxes (Supplemental Figure 6B). To test whether Slug directly represses the *LepR* promoter, we constructed mouse *LepR*–promoter luciferase-reporter plasmids and cotransfected the reporter plasmids with Slug plasmids into the hypothalamic cell line GT1-7. Slug dose-dependently suppressed *LepR*-promoter luciferase activity (Figure 7I). To test if Slug directly binds to the *LepR* promoter, we isolated the hypothalamus from WT (i.e., *Slug^+/+^*) and whole-body *Slug* knockout (i.e., *Slug^–/–^*, negative control) mice and performed ChIP-qPCR assays. *Slug^+/+^* and *Slug^–/–^* mice were fed a HFD for 14–16 weeks to increase Slug expression in *Slug^+/+^* mice. *Slug^–/–^* mice, like *Slug^ΔLepRb^* mice, were resistant to HFD-induced obesity (*Slug^+/+^*: 35.9 ± 1.13 g, n = 12; *Slug^–/–^*: 24.26 ± 0.39 g, n = 10, *P* < 0.05). We detected an abundant occupancy of hypothalamic Slug on the *LepR* promoter in *Slug^+/+^* but not *Slug^–/–^* mice (Figure 7J).

Slug has been known to regulate histone modifications in target promoters and enhancers (13), prompting us to assess histone 3 lysine-27 dimethylation (H3K27me2), H3K27 trimethylation (H3K27me3), and H3K27 acetylation (H3K27ac) in the *LepR* promoter. We placed *Slug^+/+^* and *Slug^–/–^* male mice on a HFD for 14–16 weeks, increasing hypothalamic Slug expression, and isolated the hypothalamus for ChIP-qPCR. Remarkably, *LepR* promoter H3K27me2 and H3K27me3 levels, which are repressive epigenetic marks, were significantly lower, while H3K27ac levels, which is an active epigenetic mark, were significantly higher in *Slug^–/–^* mice relative to *Slug^+/+^* mice (Figure 7K). Taken together, these results suggest that Slug directly bound to the *LepRb* promoter and epigenetically repressed LepRb expression, leading to leptin resistance.

Given that hypothalamic Slug is upregulated in obesity, we postulated that aberrant Slug might increase *LepR* promoter H3K27me2/3 levels in diet-induced obesity. We placed C57BL/6J male mice on a HFD for 10 weeks and measured H3K27me2/3 levels in the hypothalamus. *LepRb* promoter H3K27 methylations were significantly higher in HFD-fed than in chow-fed mice (Supplemental Figure 6C). HFD feeding has been reported to decrease hypothalamic LepRb expression in both mice and rats (7–9, 18). We confirmed that MBH LepR expression was lower in HFD-fed mice than in chow-fed mice (Supplemental Figure 6D). Collectively, these results suggest that Slug epigenetically suppressed *LepRb* expression, contributing to leptin resistance and obesity (Supplemental Figure 7).

## Discussion

In this study, we uncovered — as far as we know — a previously unrecognized obesity-prone action of Slug in hypothalamic neurons, particularly LepRb^+^ neurons, and provided multiple lines of genetic and physiological evidence to establish a pivotal role of hypothalamic Slug in the control of body weight and metabolism. We found that both male and female *Slug^ΔLepRb^* mice with a LepRb^+^ cell–specific deletion of *Slug* were profoundly resistant to HFD-induced, as well as age-associated, obesity, insulin resistance, glucose intolerance, and NAFLD. *Slug^ΔLepRb^* mice ate less than *Slug^fl/fl^* mice, which explains the obesity-resistant phenotype. *Slug^ΔLepRb^* mice resisted BAT whitening and maintained BAT thermogenesis on a HFD, and they recruited more beige adipocytes than *Slug^fl/fl^* mice upon cold exposure. Body core temperature was higher and cold tolerance was improved in *Slug^ΔLepRb^* mice relative to *Slug^fl/fl^* mice. Consistently, whole body energy expenditure, as assessed by O_2_ consumption and CO_2_ production and normalized to lean mass, was higher in *Slug^ΔLepRb^* mice. These observations unveil what we believe to be a previously unrecognized LepRb-neuron Slug/sympathetic nerve/adipose-thermogenesis axis. To directly demonstrate the proobesity action of hypothalamic LepRb-neuron Slug, we generated Cre-dependent, neuron-specific AAV-hSyn-DIO-Slug vectors, and showed that MBH LepRb^+^ neuron–specific overexpression of Slug was sufficient to induce obesity, glucose intolerance, insulin resistance, and NAFLD in mice on a chow diet. We confirmed these findings using a distinct Cre-dependent AAV-CAG-DIO-Slug vector. We observed that HFD feeding not only increased hypothalamic Slug expression but also promoted a conversion of hypothalamic Slug^–^ neurons to Slug^+^ neurons. We therefore consider aberrant upregulation of hypothalamic Slug as a previously unrecognized causal factor for obesity and metabolic disease.

Leptin resistance has been well documented to drive obesity progression. We found that LepRb^+^ neuron-intrinsic Slug directly induced leptin resistance. Plasma leptin levels were markedly lower in *Slug^ΔLepRb^* than in *Slug^fl/fl^* mice both before and after body weight divergence. Leptin stimulation increased hypothalamic-Stat3 phosphorylation and body-weight loss to a significantly higher level in *Slug^ΔLepRb^* than in *Slug^fl/fl^* mice on chow diet at 8–9 weeks of age, when their body weights were similar. This indicates that leptin resistance was a causal factor for, rather than a consequence of, obesity in these models. Conversely, MBH LepRb^+^ neuron-specific overexpression of Slug blunted the ability of leptin to stimulate hypothalamic-Stat3 phosphorylation and to decrease body weight. We propose that Slug-induced leptin resistance in the hypothalamus is a causal factor for obesity and its associated disorders (Supplemental Figure 7). However, these data do not exclude the possibility that Slug may induce obesity by additional mechanisms.

We demonstrated that Slug induced leptin resistance by epigenetically repressing *LepRb* transcription. We confirmed the previous reports that hypothalamic LepRb expression is decreased in diet-induced obesity (7–9, 18). It is not unexpected that reduced LepRb expression results in leptin resistance and obesity. In line with this notion, *db/+* mice with haploinsufficiency of LepRb, in certain genetic backgrounds, are prone to diet-induced obesity and metabolic disorders (19, 20). Neuronal restoration of LepRb in *db/db* mice dose-dependently reverses obesity, metabolic disorders, and fertility dysfunctions (21). Increased expression of hypothalamic LepRb is linked to resistance to diet-induced obesity and infertility in female mice (22). Notably, both mouse and human *LepRb* promoters contain putative Slug binding sites, and we verified that Slug directly bound to the *LepRb* promoter in the hypothalamus using ChIP. In cell culture, Slug directly repressed *LepRb* promoter activity. In mice, LepRb^+^ neuron-specific ablation of Slug increased — whereas MBH LepRb^+^ neuron-specific overexpression of Slug decreased — *LepRb* expression in the hypothalamus. These findings indicate that LepRb^+^ neuron-intrinsic Slug directly suppressed LepRb expression in vitro and in vivo. At the chromatin level, *Slug* deficiency decreased H3K27me2 and H3K27me3 levels, which are repressive epigenetic marks, while increasing H3K27ac levels, which is an active epigenetic mark, in the hypothalamus. Consistent with this finding, hypothalamic *LepRb* promoter H3K27me2 and H3K27me3 levels were increased in HFD-fed mice, correlated with upregulation of hypothalamic Slug. In line with these observations, Slug has been reported to bind to and recruit multiple histone methyltransferases and/or demethylases (10, 13). Based on these findings, we propose that obesogenic factors upregulate Slug in the hypothalamus. Slug recruits epigenetic modifiers to induce repressive epigenetic modifications on the *LepRb* promoter/enhancer, resulting in suppression of LepRb expression, leptin resistance, and obesity (Supplemental Figure 7). It is likely that Slug may have additional epigenetic targets involved in leptin resistance and obesity, such as positive regulators, like Sh2b1, and negative regulators, like SOCS3 and PTP1b, of LepRb signaling. Sh2b1 directly binds to JAK2 and enhances leptin action (3, 23). In contrast, SOCS3 and PTP1b inhibit LepRb signaling and induce leptin resistance (24–29). Hypothalamic Slug–elicited epigenetic reprogramming may act in concert with Sh2b1, SOCS3, PTP1b, and additional regulators to promote leptin resistance and obesity.

There are limitations in this study. Slug-associated epigenetic modifiers mediating *LepRb*-promoter histone modifications remain elusive. A cause-effect relationship between *LepRb*-promoter histone modifications and leptin resistance needs to be further confirmed. Contribution of Slug-based epigenetic reprogramming of leptin pathways to obesity needs to be quantified. Signaling pathways coupling obesogenic factors to Slug upregulation remain to be identified. HFD feeding was reported to increase, rather than decrease, LepRb expression in some hypothalamic areas (30), and the underlying mechanisms for the opposing actions of HFD on LepRb expression remain unknown. Nonetheless, this work has defined what we believe to be a new Slug-elicited epigenetic-reprogramming paradigm in the hypothalamus and laid a foundation for future studies to address these questions.

## Methods

### Animals.

*Slug^fl/fl^* and *LepRb-Cre* — *Cre* knockin in the *LepRb* 3′-UTR — mice (C57BL/6 background) were characterized previously (13, 16). *Slug^fl/fl^* mice were crossed with *LepRb-Cre* mice to generate *Slug^ΔLepRb^* mice — *Slug^fl/fl^;Cre^+/+^*. Because Cre expression is insufficient in *LepRb-Cre^+/–^* mice to delete the target genes, we generated homozygous *LepRb-Cre^+/+^* to delete *Slug* following the previously established protocols (31). We report that homozygous *LepRb-Cre^+/+^* mice are normal, and their body weight is that of WT mice (3). Mice were housed on a 12-hour light/12-hour dark cycle at an ambient temperature of 25^0^C and fed ad libitum a normal chow diet with 9% fat (TestDiet) or a HFD with 60% fat (Research Diets).

### AAV-hSyn-DIO-Slug and AAV-CAG-DIO-Slug vectors and hypothalamic LepRb^+^ neuron-specific overexpression of Slug.

The *Slug* cDNA sequences were flanked by 2 identical loxp sites at its 5′ and the 3′ ends in a head-to-head orientation. The DIO *Slug* cDNA was inserted into the 3’ end of the hSyn promoter (AAV-hSyn-DIO-Slug) or the CAG promoter (AAV-CAG-DIO-Slug) (Supplemental Figure 3, A and D). Cre is required for expression of recombinant Slug by inverting *Slug* cDNA orientation. To generate mice with hypothalamic LepRb^+^ neuron-specific overexpression of Slug, 8-week-old *Slug^ΔLepRb^* (*Slug^fl/fl^;Cre^+/+^*) and *Slug^fl/fl^* (control) male mice were isoflurane-anesthetized and mounted on an Ultra Precise Small Animal Stereotaxic Alignment System (David KOPF Instruments). A small opening was made in the skull. AAV-hSyn-DIO-Slug or AAV-hSyn-DIO-mCherry vectors (0.5 μL were bilaterally injected into the MBH (–1.5 mm anterior-posterior ± 0.4 mm medial-lateral and –5.8 dorsal-ventral) using UltraMicroPumps with SYS-Micro4 Controller (UMP3-2, World Precision Instruments Inc.). In a separate cohort, 9-week-old male *Slug^ΔLepRb^* (*Slug^fl/fl^;Cre^+/–^*) mice were bilaterally microinjected with AAV-CAG-DIO-Slug or AAV-CAG-DIO-mCherry vectors into the MBH. AAV-transduced mice were placed on a chow diet and subjected to various tests as described in the figure legends.

### Leptin stimulation of weight loss.

Mice were i.p. injected with leptin (0.25 mg/kg body weight) twice — once at 6 pm and again at 12 am — daily for 3–4 days, and body weight was recorded.

### Cold-tolerance test.

Empty cages with no bedding materials were precooled at 4°C in a rodent environmental chamber (RIS33SD, Innovative Solutions). Mice were fasted overnight, transferred to the precooled cage, and housed individually. They had free access to water, but there was no food for them during the cold-exposure experiment. Core body temperature was measured hourly via the rectum.

### Plasma insulin and leptin measurement, GTT, and ITT.

Blood samples were collected from tail veins. Plasma insulin and leptin were measured using insulin and leptin ELISA kits (Crystal Chem), respectively. For GTT, mice were fasted overnight and i.p. injected with glucose (2 g/kg body weight), and blood glucose was measured 0, 15, 30, 60, and 120 minutes after injection. For ITT, mice were fasted for 6 hours and i.p. injected with insulin (0.75 U/kg).

### Fat content and energy expenditure.

Fat content and lean body mass (normalized to body weight) were measured using a dual-energy X-ray absorptiometry pDexa (Norland Stratec). Energy expenditure was measured by indirect calorimetry using the Windows Oxymax Equal Flow System (Columbus Instruments). Volume of O_2_ consumption (VO_2_) and volume of CO_2_ production (VCO_2_) were normalized to body lean mass. Additionally, ANCOVA analysis was performed following instructions by the National Institute of Diabetes and Digestive and Kidney Diseases (NIDDK) Mouse Metabolic Phenotyping Centers (www.MMPC.org/shared/regression.aspx). Data were analyzed using linear regression analysis to assess the impact of covariates on energy expenditure. Average VO_2_ and VCO_2_ values were used for ANCOVA, and either lean mass or body weight were set as covariates.

### Liver TAG levels.

Liver samples were homogenized in 1% acetic acid and extracted by chloroform-methanol (2:1). The organic phase was dried by evaporation and dissolved in isopropanol. TAG levels were measured using a TAG assay kit (Pointe Scientific Inc.) and normalized to liver weight.

### Immunostaining and RNAscope fluorescence assays.

Frozen brain, BAT, and liver sections were cut using Leica cryostat (Leica Biosystems Nussloch GmbH), and immunostained with appropriate antibodies (Supplemental Table 1). H&E staining of WAT and liver samples was performed on paraffin sections. RNAscope fluorescence assays were performed by the In Situ Hybridization Lab, Microscopy, Imaging, and Cellular Physiology Core, Michigan Diabetes Research Center. Slug probes hybridized the target *Slug* mRNA sequences that were deleted in *Slug^ΔLepRb^* mice. Hypothalamic frozen sections were prepared and used for RNAscope fluorescence assays following manufacturer’s instructions and using LepR probes, Slug probes, and RNAscope multiplex fluorescence detection reagents V2 kits (Advanced Cell Diagnostics Inc. [ACD]). Images were obtained using BX51 Microscope coupled with a DP72 digital camera (Olympus).

### Immunoprecipitation and immunoblotting.

Tissues or cell cultures were homogenized in ice-cold lysis buffer (50 mM Tris HCl, pH 7.5, 0.5% Nonidet P-40, 150 mM NaCl, 2 mM EGTA, 1 mM Na_3_VO_4_, 100 mM NaF, 10 mM Na_4_P_2_O_7_, 1 mM phenylmethylsulfonyl fluoride, 10 μg/ml aprotinin, 10 μg/mL leupeptin). Tissue or cell extracts were immunoprecipitated and immunoblotted with appropriate antibodies (Supplemental Table 1).

### ChIP.

Hypothalamic or cell culture samples were treated with 1% formaldehyde for 10 minutes. Genomic DNA was extracted and sheared to 200–500 bp fragments using a sonicator (QSONICA). DNA-protein complexes were immunoprecipitated with the appropriate antibodies (Supplemental Table 1). Crosslinking was reversed by heating at 65^°^C for 4 hours. DNA was recovered using commercial kits or chemical purifications and used for qPCR analysis using *LepR* 5′- and 3′-primers (Supplemental Table 2).

### qPCR.

Total RNAs were extracted from cells, tissues, and ChIP supernatant samples using TRIzol reagent (Invitrogen). The first-strand cDNAs were synthesized using random primers and M-MLV reverse transcriptase (Promega). qPCR was performed using Radiant SYBR Green 2X Lo-ROX qPCR Kits (Alkali Scientific), a StepOnePlus RT PCR Systems (Life Technologies Corporation), and appropriate primers (Supplemental Table 2).

### Affymetrix microarray analysis.

The hypothalamus was isolated from *Slug^ΔLepRb^* and *Slug^fl/fl^* males at 10 weeks of age. Total hypothalamic RNAs were extracted and analyzed using Affymetrix GeneChips (the University of Michigan DNA sequencing core). Variable transcripts were analyzed using Ingenuity Pathway Analysis (QIAGEN). The GeneChips data sets were deposited in Gene Expression Omnibus (GEO) (GSE217748).

### LepR luciferase reporter assays.

The mouse *LepR* promoter (from –863 to +111) was isolated by PCR and inserted into pGL3 vectors. GT1-7 cells, provided by Malcolm J. Low (University of Michigan, Ann Arbor, Michigan, USA) were grown in DMEM containing 5 mM glucose and 10% FBS at 5% CO_2_ and 37°C. GT1-7 cells were transiently cotransfected with *pGL3-LepR* luciferase reporter plasmids and appropriate other expression vectors, using polyethylenimine (Sigma-Aldrich). Luciferase activity was measured 48 hours after transfection using a kit (Promega) and normalized to β-gal internal control.

### Data availability.

The authors declare that the data supporting the findings of this study are available within the article and supplemental files.

### Statistics.

Data were presented as mean ± SEM. Differences between 2 groups were analyzed by 2-tailed Student’s *t* test. Comparisons between more than 2 groups or variables were analyzed by 1-way or 2-way ANOVA and/or Tukey’s post hoc test using GraphPad Prism 8. A *P* value of less than 0.05 was considered significant.

### Study approval.

Animal research complied with all relevant ethnic regulations. Animal experiments were conducted following the protocols approved by the University of Michigan IACUC.

## Author contributions

MHK, YL, QZ, and LJ conducted experiments; MHK and LR designed experiments and wrote the manuscript; and MHK, MGM, WW, and LR edited the manuscript.

## Supplementary Material

Supplemental data

Supplemental data set 1

## Version 1 12/13/2022

In-Press Preview

## Version 2 02/15/2023

Electronic publication
