# Supplementary material for: LepRb+ cell–specific deletion of Slug mitigates obesity and nonalcoholic fatty liver disease in mice
Source: J Clin Invest. 2023 Feb 15;133(4):e156722. doi: 10.1172/JCI156722 (PMC9927931; doi:10.1172/JCI156722)

Supplemental Materials for

LepRb cell-specific deletion of *Slug* mitigates obesity and NAFLD

Min-Hyun Kim[1§], Yuan Li[1], Qiantao Zheng[1], Lin Jiang[1], Martin G. Myers Jr[1,2], Wen-Shu Wu[3], Liangyou Rui[1,4,#]

[1]Department of Molecular & Integrative Physiology, [2]Division of Metabolism and Endocrinology, Department of Internal Medicine, University of Michigan Medical School, Ann Arbor, Michigan 48109, USA, [3]Division of Hematology/Oncology, Department of Medicine, UI Cancer Center, University of Illinois at Chicago, Chicago, IL 60612, USA, [4]Division of Gastroenterology and Hepatology, Department of Internal Medicine, University of Michigan Medical School, Ann Arbor, Michigan 48109, USA.

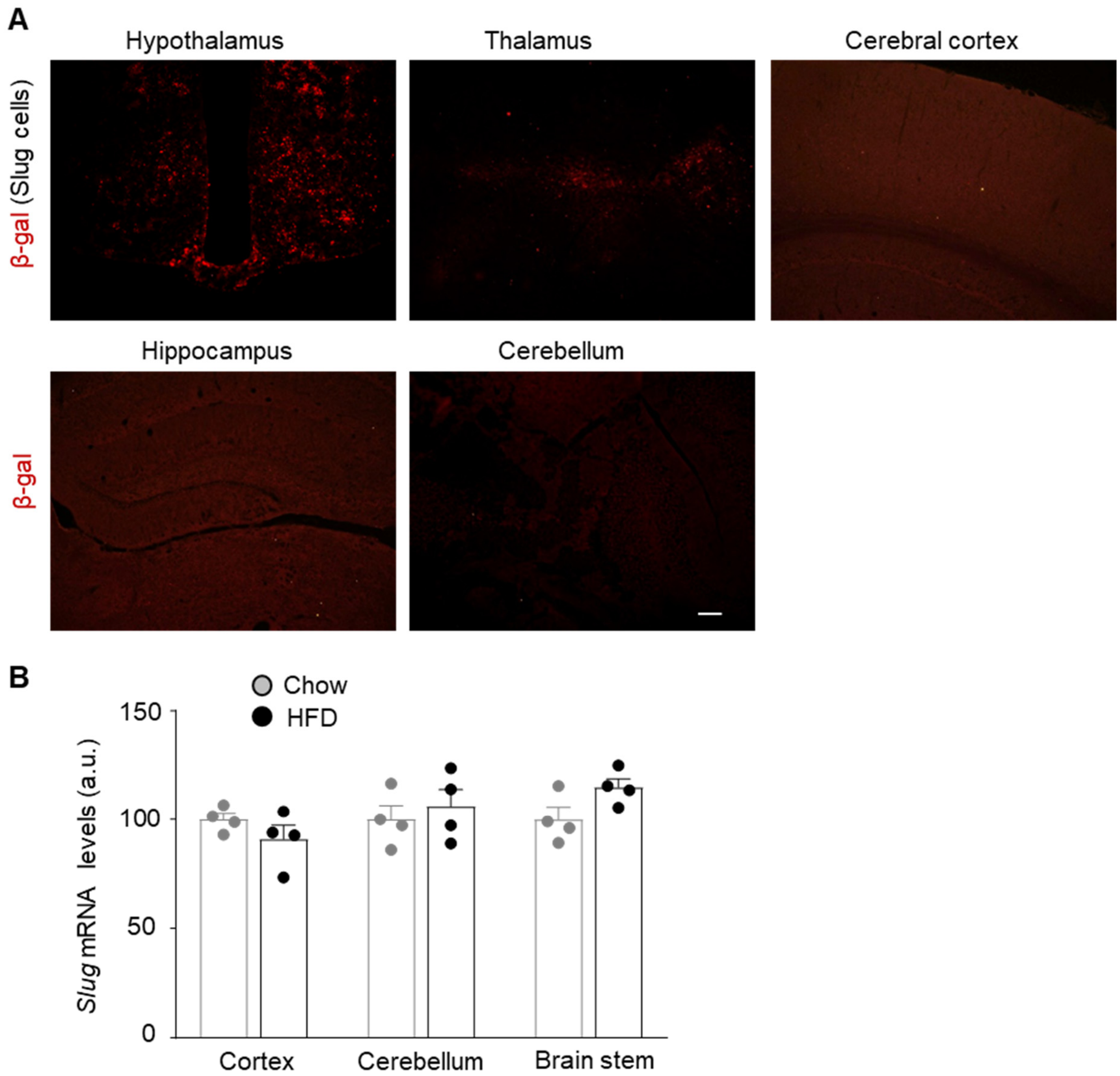

Supplemental Figure 1. Brain Slug expression. (A) Brain sections were prepared from *Slug*$^{LacZ/+}$ mice and immunostained with anti-β-gal antibody to visualize Slug-expressing cells. Scale bar: 200 µm. **(B)** C57BL/6J male mice (7 weeks) were fed a chow diet or HFD for 16 weeks. *Slug* mRNA levels were measured in various brain areas by qPCR and normalized to 36B4 expression (n=4 mice per group). Data are presented as mean ± SEM.

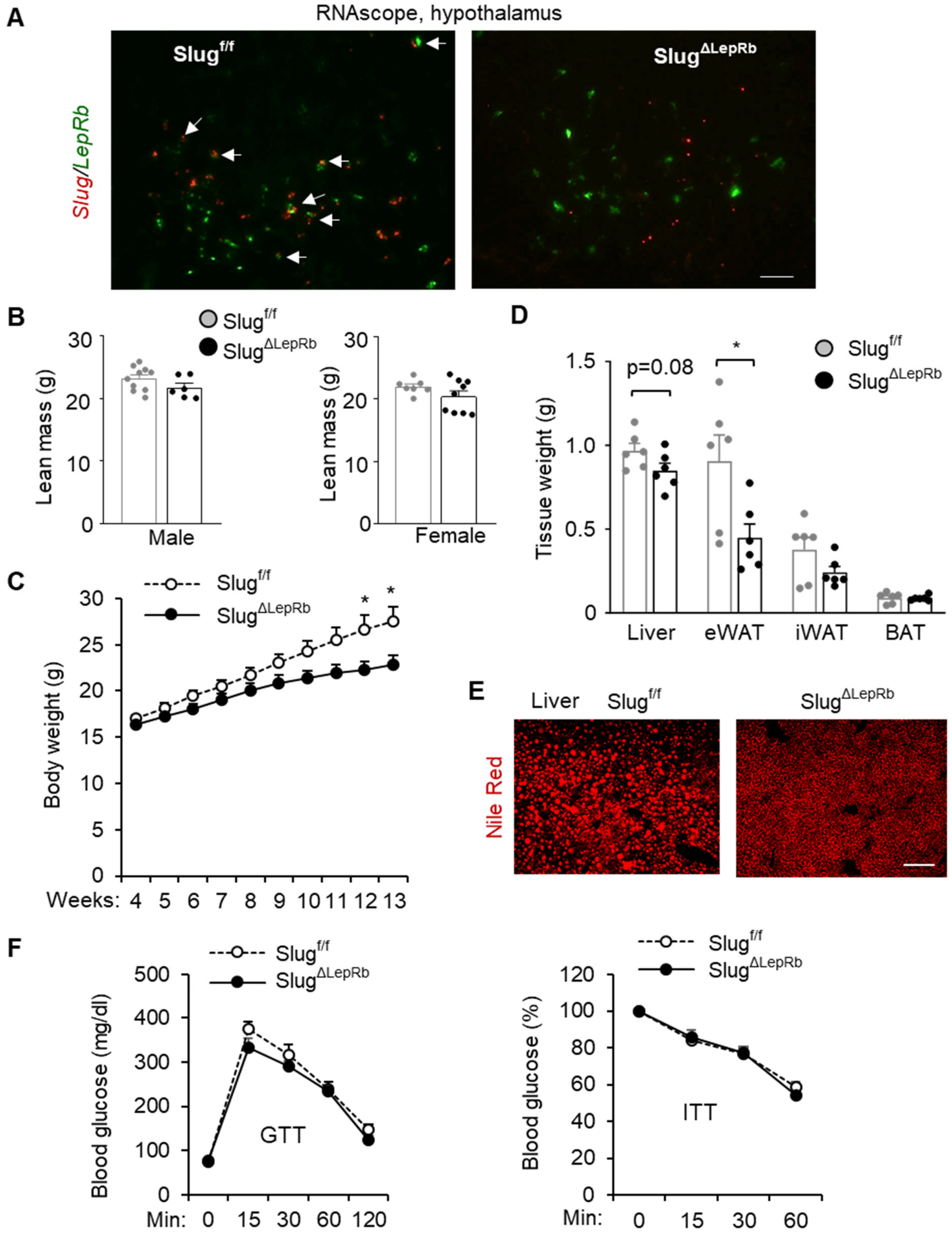

Supplemental Figure 2. LepRb cell-specific deletion of *Slug* is associated with resistance to obesity and NAFLD. (A) Brain sections were prepared from $Slug^{f/f}$ (n=3) and $Slug^{\Delta LepRb}$ (n=3) male mice at 8 weeks of age on chow diet. Hypothalamic *Slug* and *LepRb* mRNAs were detected using RNAscope assays. The Slug probes bind to the mRNA sequences encoded by the deleted *Slug* DNA sequences in $Slug^{\Delta LepRb}$ mice. Scale bar: 20 µm. **(B)** Lean mass was measured by pDEXA at 10 weeks of age. Male $Slug^{f/f}$: n=10, male $Slug^{\Delta LepRb}$: n=6, female $Slug^{f/f}$: n=7, female $Slug^{\Delta LepRb}$: n=9. **(C-**

D) *Slug*$^{f/f}$ (n=6) and *Slug*$^{\Delta LepRb}$ (n=6) male mice were fed a standard chow diet. **(C)** Body weight. **(D)** Tissue weight at 13 weeks of age. **(E)** Representative Nile red staining of liver sections in males on HFD for 13 weeks (n=3 mice per genotype). Scale bar: 200 μm. **(F)** *Slug*$^{f/f}$ (n=6) and *Slug*$^{\Delta LepRb}$ (n=6) male mice were fed a standard chow diet. GTT and ITT were performed at 8 weeks of age. Data are presented as mean ± SEM. *p<0.05, 2-tailed unpaired Student's *t* test (B and D) and two-way ANOVA (C, F).

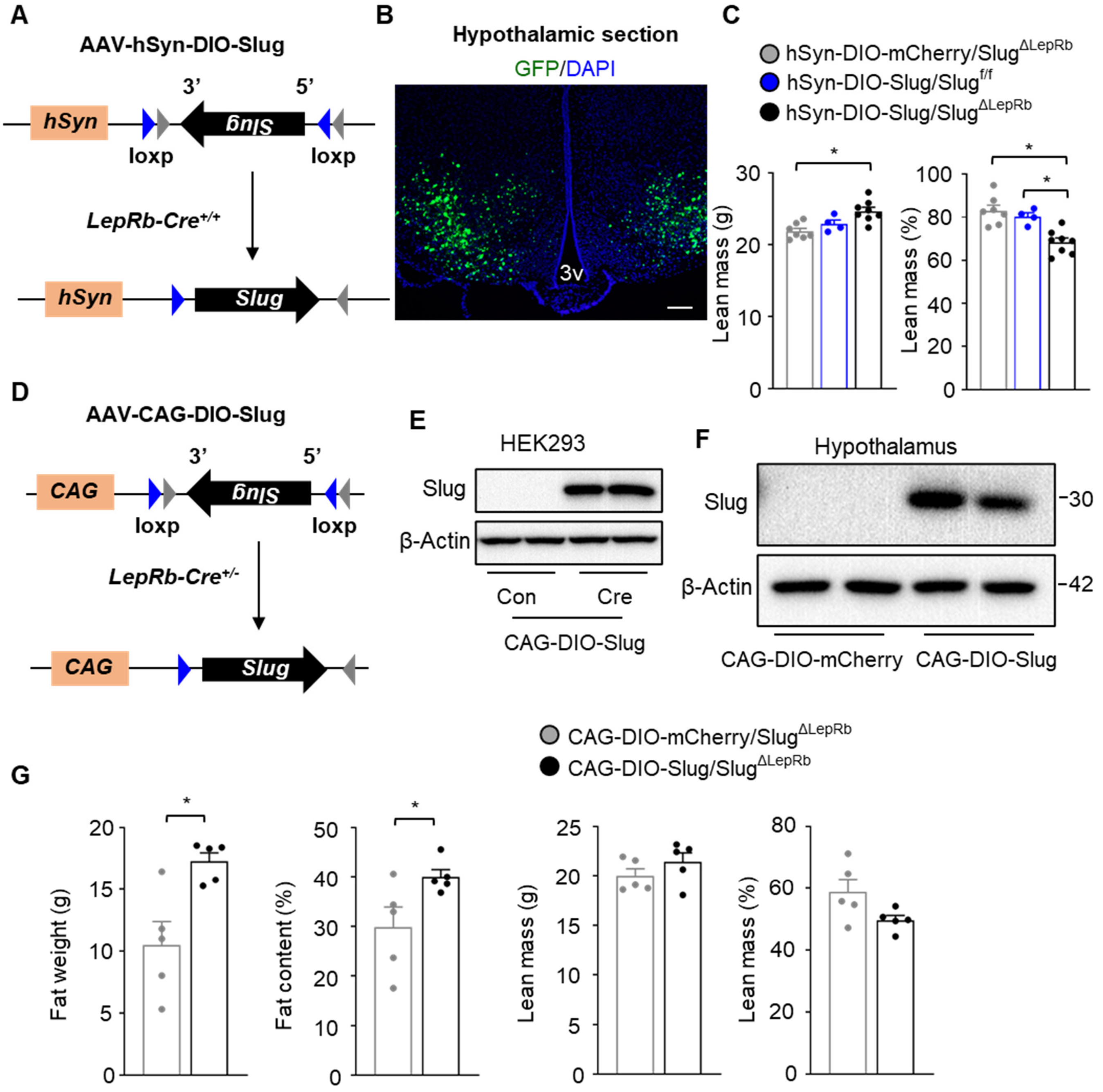

Supplemental Figure 3. Cre-dependent overexpression of Slug in hypothalamic LepRb neurons induces obesity. (A) Schematic representation of AAV-hSyn-DIO-Slug vectors and Cre-dependent expression of Slug. **(B)** AAV-CAG-GFP vectors were bilaterally microinjected into the MBH of C57BL/6J mice. Hypothalamic sections were prepared 3 weeks post transduction. GFP was detected by fluorescent microscope. **(C).** AAV-hSyn-DIO-Slug (n=8) or AAV-hSyn-DIO-mCherry (n=7)

vectors were bilaterally microinjected into the MBH of *Slug*$^{\Delta LepRb}$ (*Slug*$^{f/f}$*;LepRb-Cre*$^{+/+}$) males at 8 weeks of age on chow diet. As an additional control, AAV-hSyn-DIO-Slug vectors were bilaterally microinjected into the MBH of *Slug*$^{f/f}$ males at 8 weeks of age on chow diet (n=4). Lean mass was measured by pDexa and normalized to body weight in 11 weeks post AAV transduction. **(D)** Schematic representation of AAV-CAG-DIO-Slug vectors and Cre-dependent expression of Slug. **(E)** AAV-CAG-DIO-Slug plasmids were cotransfected into HEK293 cells with Cre or empty (Con) expression plasmids. Cell extracts were prepared 2 days later and immunoblotted with the indicated antibodies. **(F)** AAV-CAG-DIO-Slug or AAV-CAG-DIO-mCherry vectors were bilaterally microinjected into the MBH of *Slug*$^{\Delta LepRb}$ (*Slug*$^{f/f}$*;LepRb-Cre*$^{+/-}$) male mice at 9 weeks of age on chow diet. Hypothalamic extracts were prepared in 10 weeks later and immunoblotted with antibodies against Slug or β-actin. **(G)** AAV-CAG-DIO-Slug or AAV-CAG-DIO-mCherry vectors were bilaterally microinjected into the MBH of *Slug*$^{\Delta LepRb}$ (*Slug*$^{f/f}$*;LepRb-Cre*$^{+/-}$) male mice at 9 weeks of age on chow diet. Fat weight, fat content (normalized to body weight), and lean mass were measured in 9 weeks after AAV transduction using pDexa (n=5 mice per group). Data are presented as mean ± SEM. *$p<0.05$, 2-tailed unpaired Student's *t* test (F) and one-way ANOVA (C).

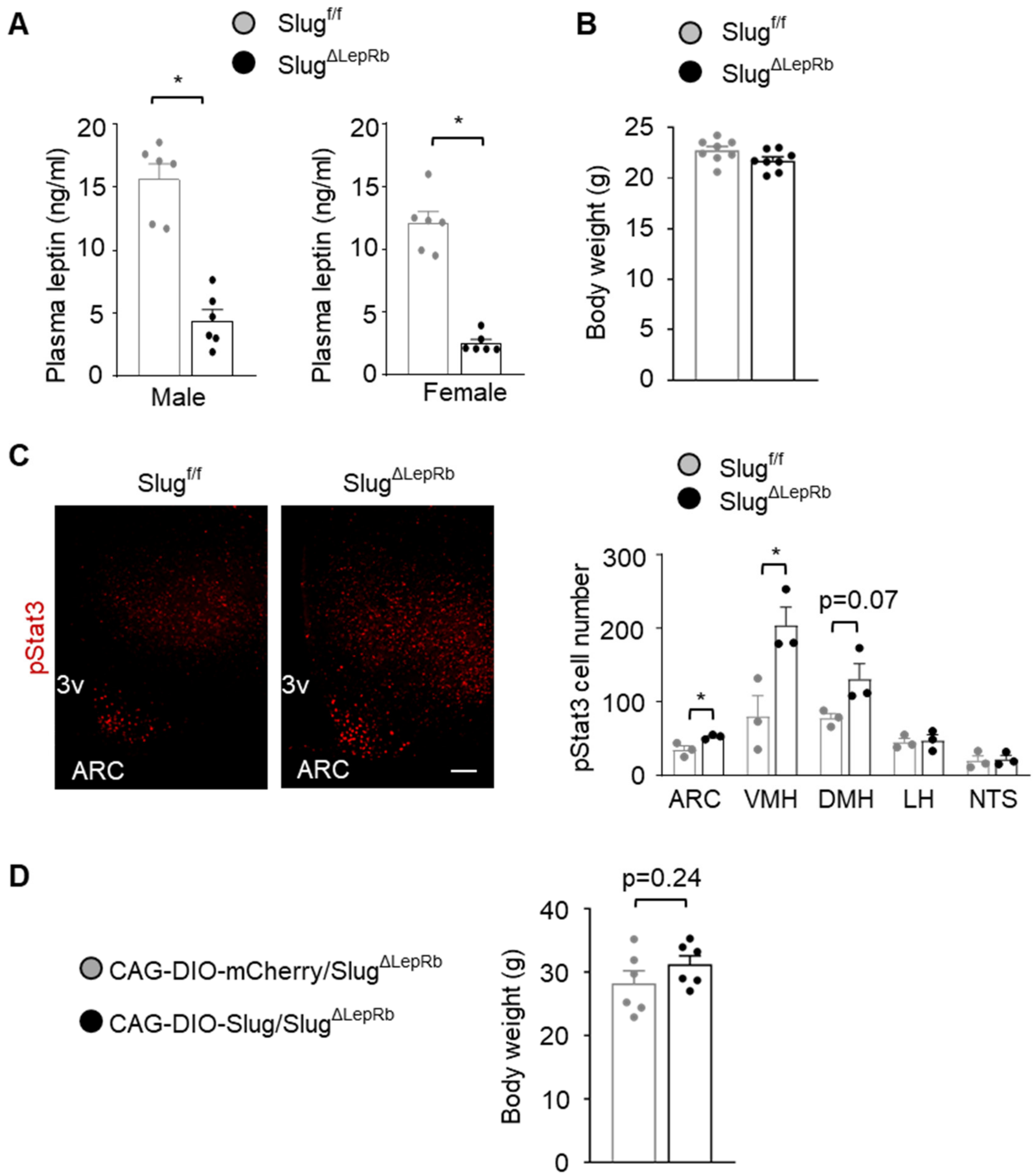

Supplemental Figure 4. Ablation of Slug in LepRb neurons increases leptin sensitivity. (A) Overnight-fasted plasma leptin levels after 12 weeks on HFD (n=6 mice per group). (**B**) Body weight at 8 weeks of age on chow diet (n=8 male mice per group). (**C**) *Slug*$^{f/f}$ and *Slug*$^{\Delta LepRb}$ males (7 weeks) were treated with leptin (icv, 0.1 µg/mouse) for 20 min. Hypothalamic sections were immunostained with anti-phospho-Stat3 antibody. Phopho-Stat3 neurons were counted in the indicated areas (n=3 mice per group). LH: lateral hypothalamus, NTS: nucleus tractus solitarius. Scale bar: 200 µm. **(D)** AAV-CAG-DIO-Slug or AAV-CAG-DIO-mCherry vectors were bilaterally microinjected into the MBH of *Slug*$^{\Delta LepRb}$ (*Slug*$^{f/f}$*;LepRb-Cre*$^{+/-}$) male mice at 9 weeks of age on chow diet. Body weight was measured in 2 weeks after AAV transduction (n=6 mice per group). Data are presented as mean ± SEM. *p<0.05, 2-tailed unpaired Student's *t* test.

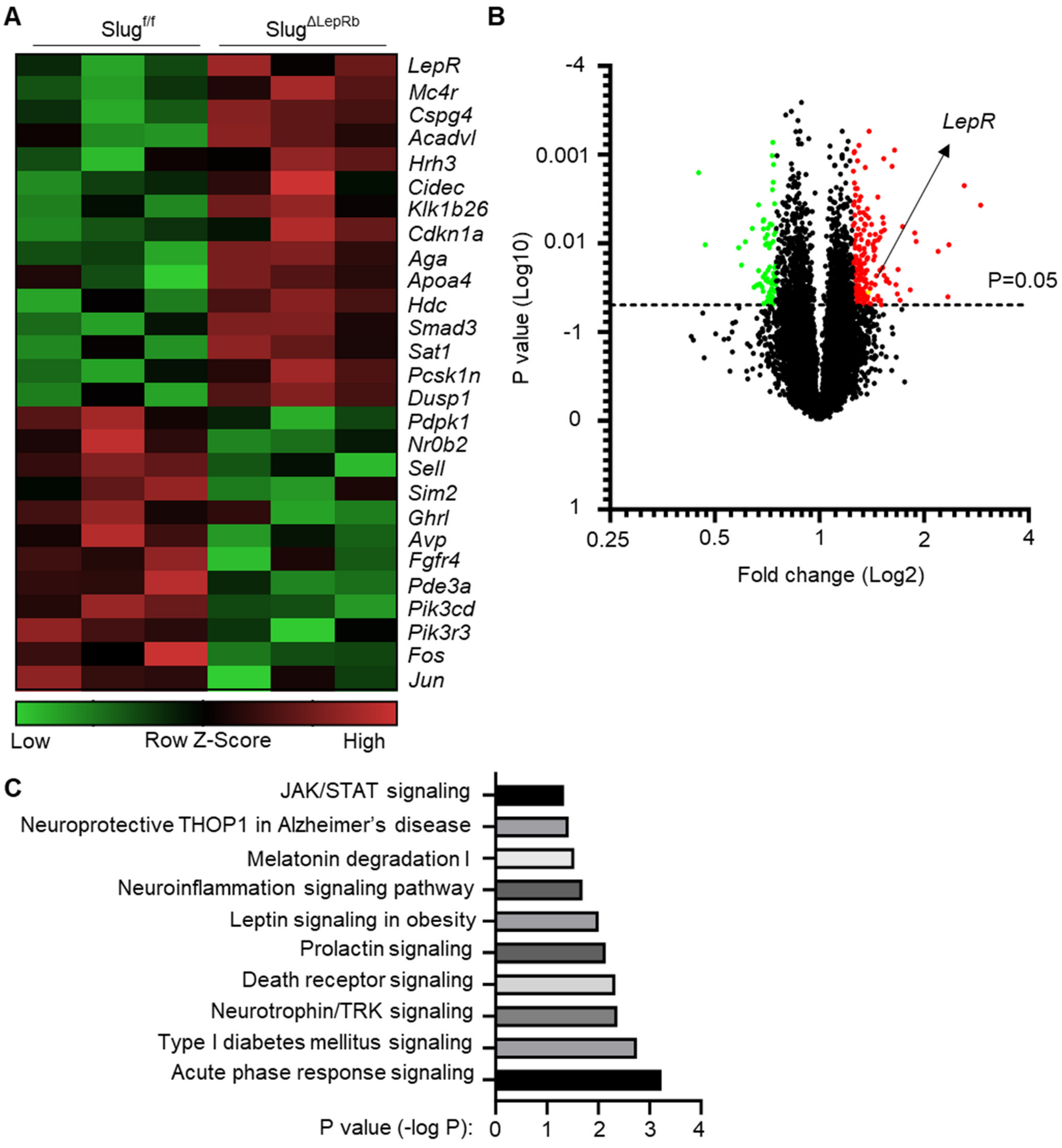

Supplemental Figure 5. Hypothalamic gene expression profiles in *Slug*$^{f/f}$ and *Slug*$^{\Delta LepRb}$ Mice. Hypothalamic RNAs were extracted from *Slug*$^{f/f}$ (n=3) and *Slug*$^{\Delta LepRb}$ (n=3) males at 8 weeks of age and subjected to Affymetrix microarray analysis (the University of Michigan DNA sequencing core). The gene expression dataset is deposited in a public repository. **(A)** Heatmap presentation of hypothalamic gene expression (Graphpad Prism 8). **(B)** A volcano plot of hypothalamic gene expression (Graphpad Prism 8). Red: fold change >1.25, p <0.05; green: fold change <0.75, p <0.05. **(C)** Pathway analysis using QIAGEN Ingenuity Pathway Analysis software.

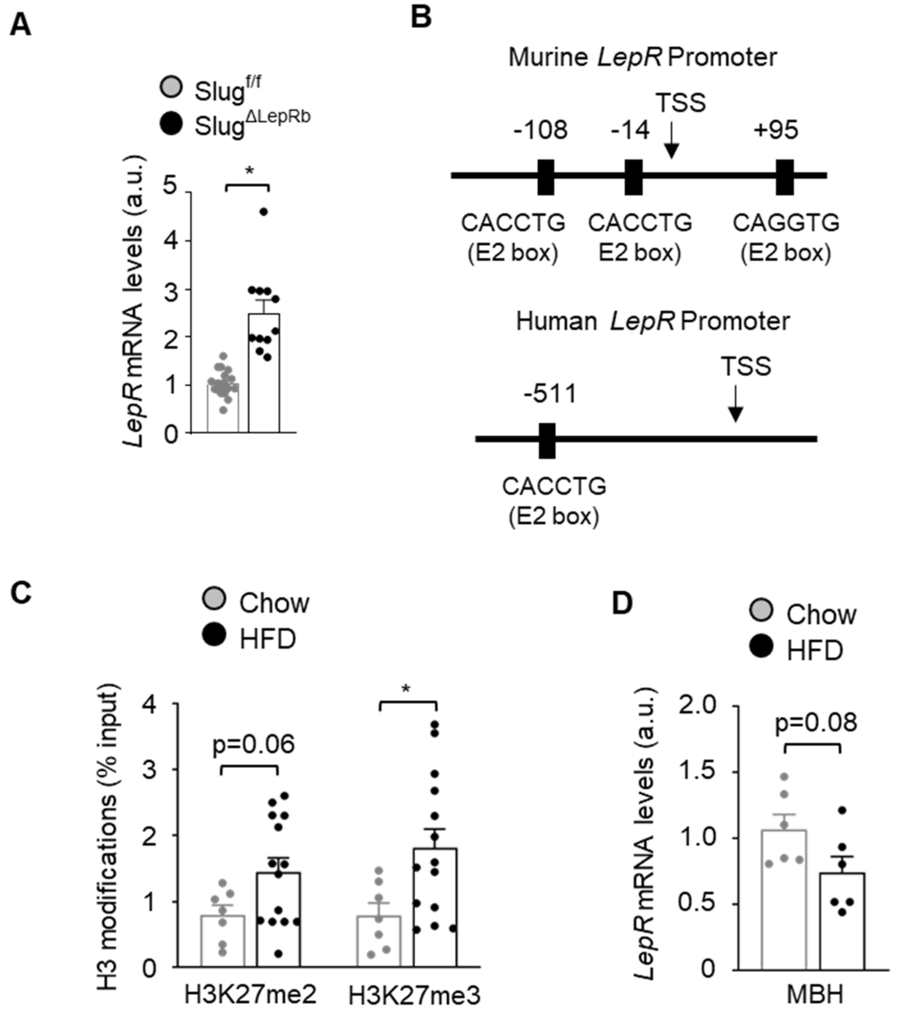

Supplemental Figure 6. Slug binding sites in the *LepRb* promoter and HFD-induced increase in hypothalamic *LepRb* promoter H3K27 methylation. (A) Male hypothalamic *LepR* mRNA levels (normalized to 36B4 levels, HFD for 15 weeks). $Slug^{f/f}$: n=18, $Slug^{\Delta LepRb}$: n=11. **(B)** Putative Slug binding sites (E2 boxes) in murine and human *LepR* promoters. TSS: transcription start site. **(C-D)** C57BL/6J male mice (7 weeks) were fed a HFD or a chow diet for 10 weeks. **(C)** Hypothalamus was harvested and hypothalamic *LepR* promoter H3K27me2 and H3K27me3 levels were measured using ChIP-qPCR. Chow: n=7 mice, HFD: n=14 mice. **(D)** The MBH was isolated and MBH *LepR* mRNA levels were measured by qPCR (normalized to 36B4 levels, n=6 mice per group). Data are presented as mean ± SEM. *p<0.05, 2-tailed unpaired Student's *t* test.

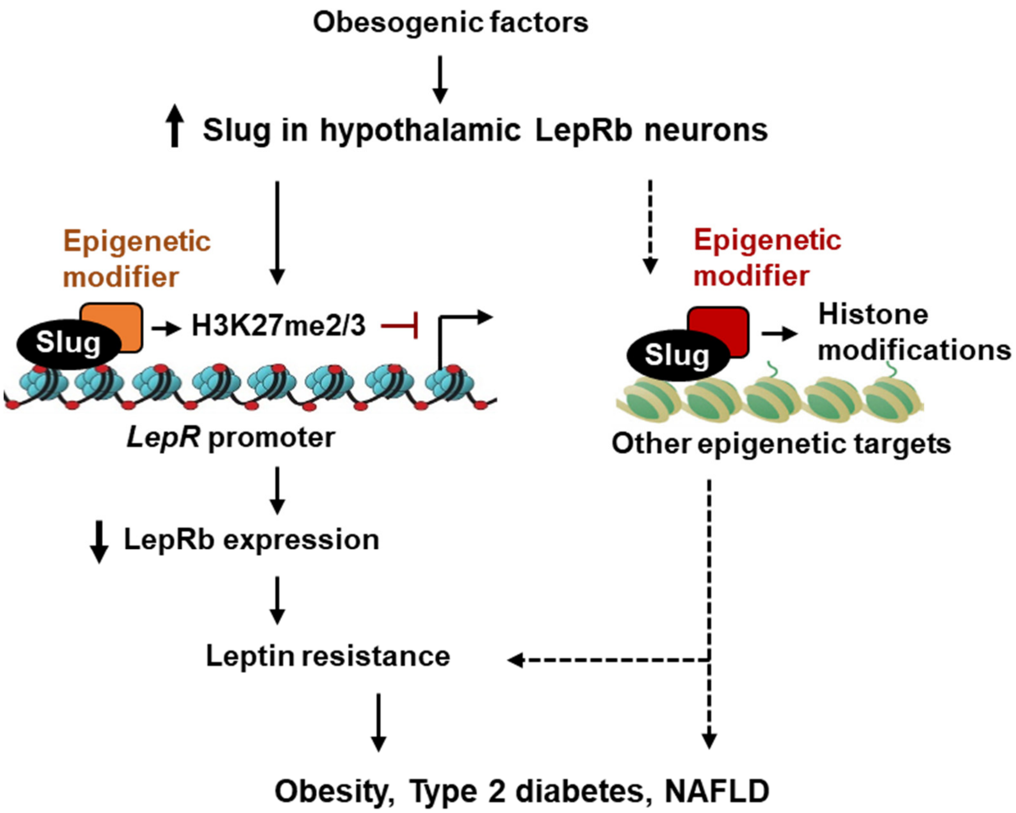

Supplemental Figure 7. Hypothalamic Slug promotes obesity by an epigenetic mechanism. Obesogenic molecules and pathways stimulate Slug expression in the hypothalamus, particularly in LepRb neurons. Slug in turn recruits epigenetic modifiers to catalyze histone modifications on LepR promoter and other target promoters, thereby epigenetically reprogramming hypothalamic energy balance circuits. Slug-induced H3K27 methylations suppress LepRb expression, leading to leptin resistance and obesity. Hypothalamic Slug promotes obesity by both LepRb expression-dependent and -independent mechanisms.

Supplemental Table 1. Antibody list.

ANTIBODY	SOURCE	Cat #	RRID	DILUTION
Ucp-1	EMD Millipore	662045	RRID:AB_212605	1:10,000
Akt	Cell Signaling Technology	4691	RRID:AB_915783	1:15,000

Tyrosine hydroxylase	Santa Cruz	sc-14007	RRID:AB_671397	1:500
pAkt (pThr308)	Cell Signaling Technology	4056	RRID:AB_331163	1:5,000
pAkt (pSer473)	Cell Signaling Technology	4060	RRID:AB_2315049	1:5,000
pStat3 (pTyr705)	Santa Cruz	sc-8059	RRID:AB_628292	1:5,000
pStat3 (pTyr705) (IF)	Cell Signaling Technology	9145	RRID:AB_2491009	1:300
Stat3	Santa Cruz	sc-8019	RRID:AB_628293	1:5,000
α-Tubulin	Santa Cruz	sc-5286	RRID:AB_628411	1:5,000
β-gal	Abcam	ab9361	RRID:AB_307210	1:1,000
NeuN	Cell Signaling Technology	12943	RRID:AB_2630395	1:10,000
Slug	Abcam	ab27568	RRID:AB_777968	1:2,000
GFAP	NeuroMab	75-240	RRID:AB_10672299	1:5,000
Slug (ChIP)	Cell Signaling Technology	9585	RRID:AB_2239535	1:100
H3K4me2	Cell Signaling Technology	9725	RRID:AB_10205451	1:100
H3K9me2	Cell Signaling Technology	4658	RRID:AB_10544405	1:100
H3K27me2	Cell Signaling Technology	9728	RRID:AB_1281338	1:100
H3K27me3	Cell Signaling Technology	9733	RRID:AB_2616029	1:100
H3K27ac	EMD Millipore	07-360	RRID:AB_310550	1:100
β-actin	Abclonal	AC026	RRID:AB_2768234	1:100,000

Supplemental Table 2. qPCR primer list.

**Genes**	**Forward**	**Reverse**
Ucp-1	ATACTGGCAGATGACGTCCC	GTACATGGACATCGCACAGC
36B4	AAGCGCGTCCTGGCATTGTCT	CCGCAGGGGCAGCAGTGGT
Slug	ATTGCCTTGTGTCTGCAAGAT	TTTTGGAGCAGTTTTTGCACT
Ucp1	ATACTGGCAGATGACGTCCC	GTACATGGACATCGCACAGC
LepR	TGTTTTGGGACGATGTTCCA	AAAGATGCTCAAATGTTTCAGGC
*Pgc1*α	TGGACGGAAGCAATTTTTCA	TTACCTGCGCAAGCTTCTCT
Dio2	GCACGTCTCCAATCCTGAAT	TGAACCAAAGTTGACCACCA
*PPAR*γ	CCAGAGTCTGCTGATCTGCG	GCCACCTCTTTGCTCTGATC
Prdm16	AGCAGCTGAGGAAGCATTT	GCGTGGAGAGGAGTGTCTTC
LepR (ChIP)	CTAGATGCAGGAATGCCCTCT	TCCGGGACTTAAGGGGTTGA

Full unedited gel for Fig 3E

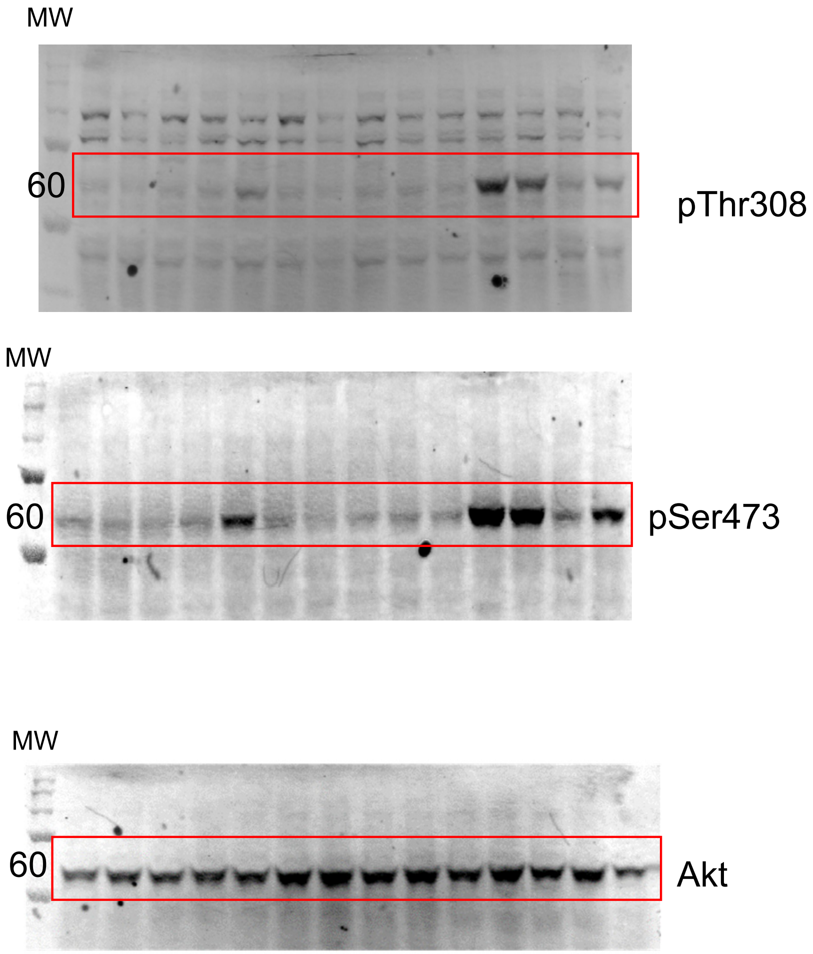

Full unedited gel for Fig 6A

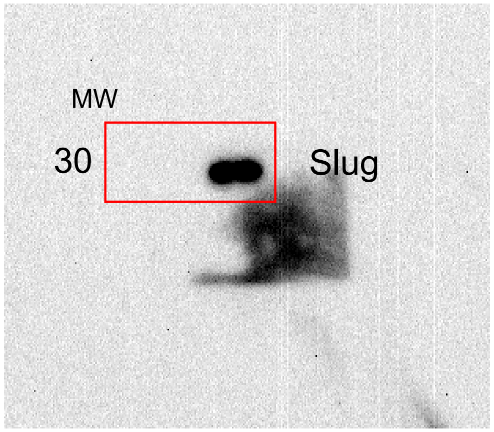

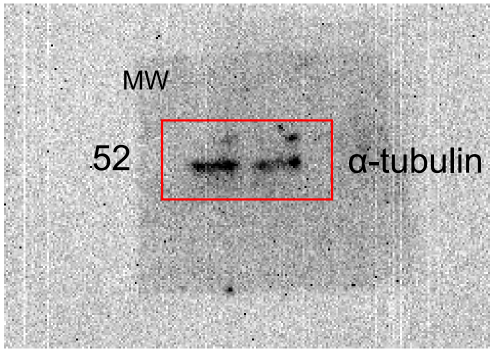

Full unedited gel for Fig 6B

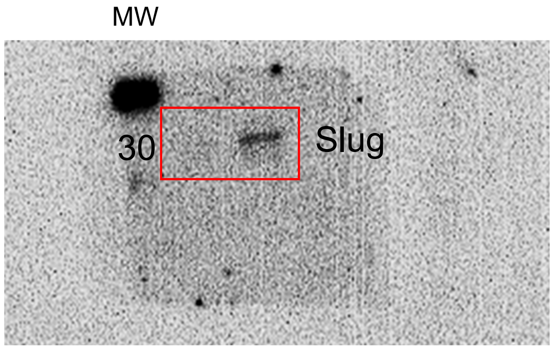

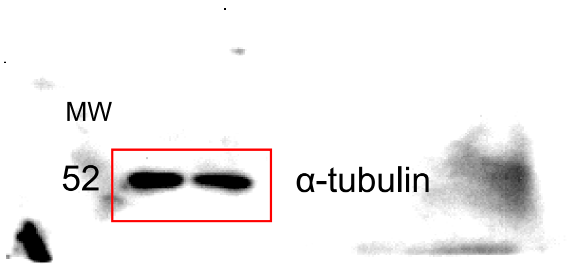

Full unedited gel for Fig 7D

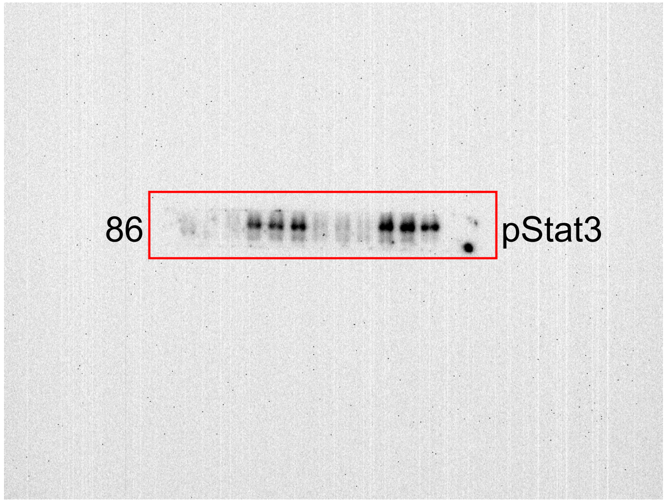

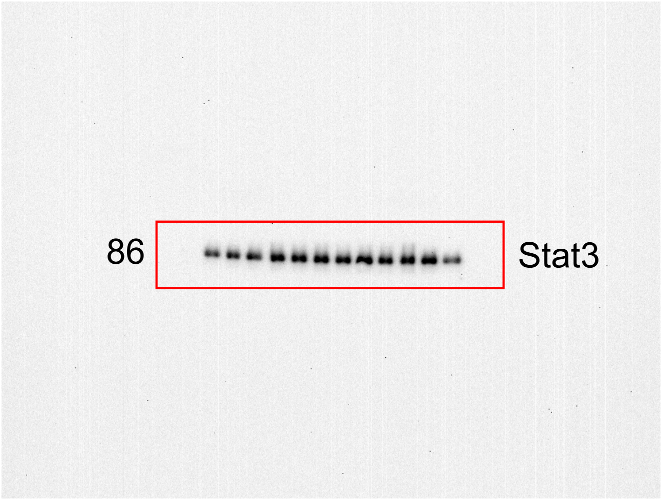

Full unedited gel for Fig 7F

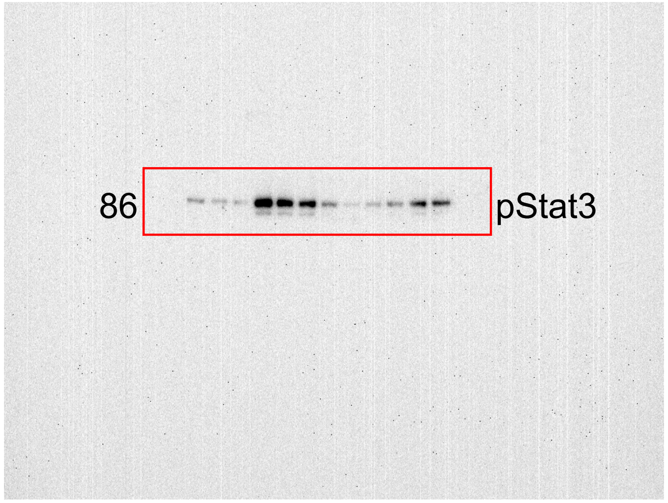

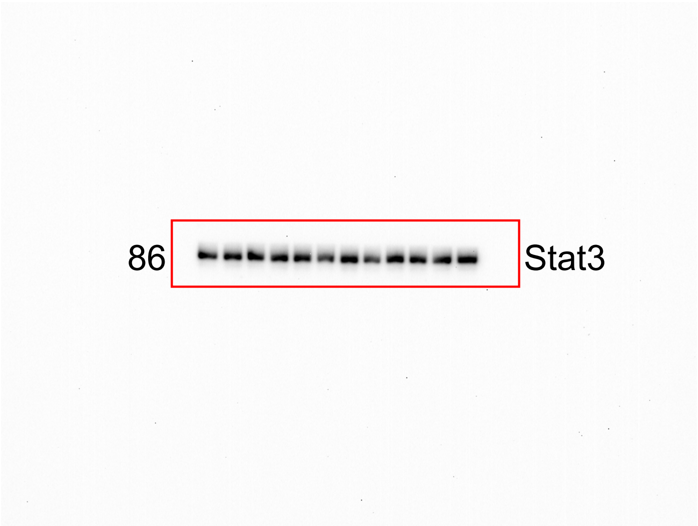

Full unedited gel for Fig S3E

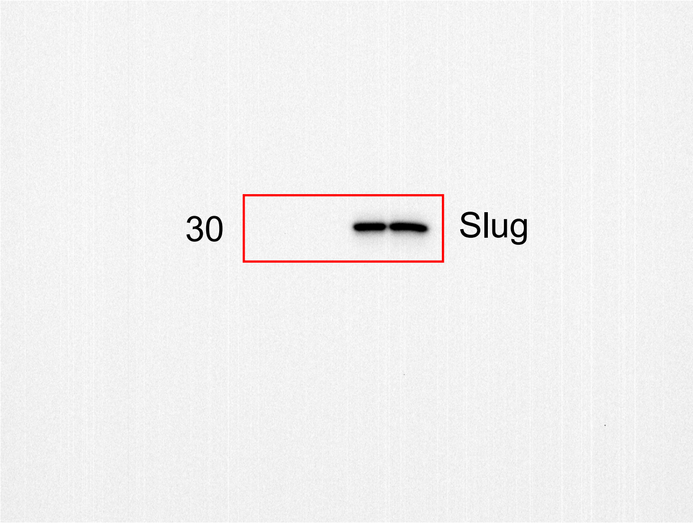

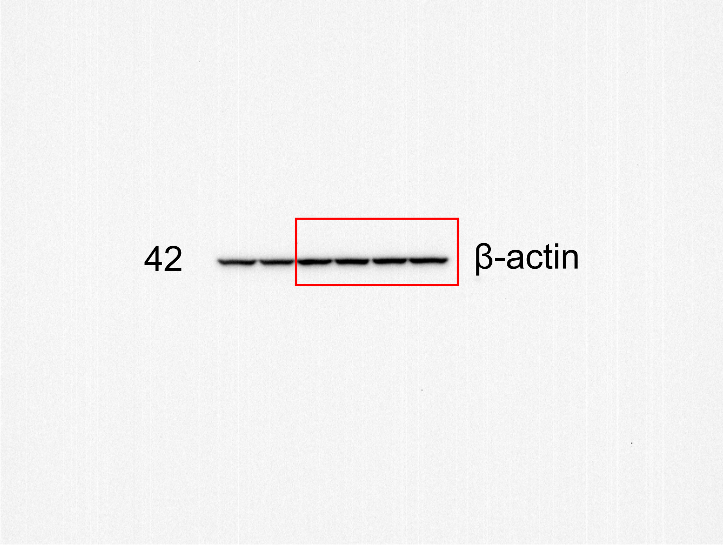

Full unedited gel for Fig S3F

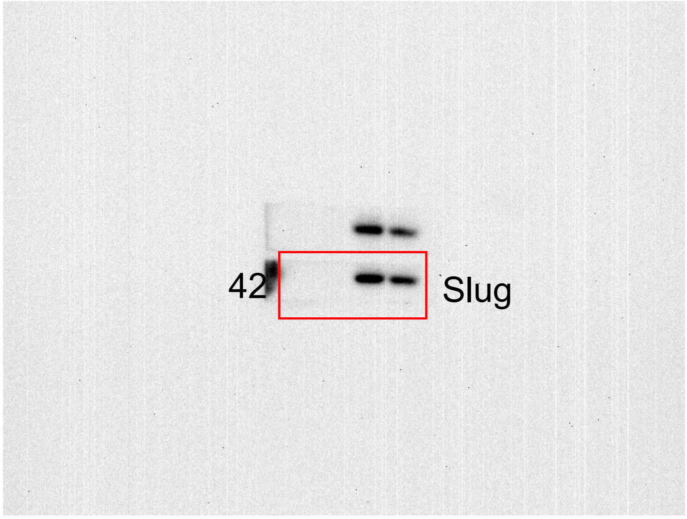

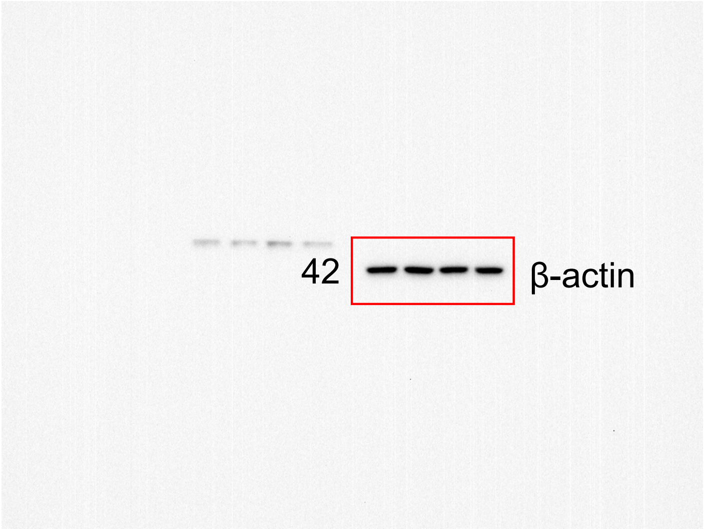

Supplement: Supplemental data [file jci-133-156722-s116.pdf]
